# A single mutation increases heavy-chain heterodimer assembly of bispecific antibodies by inducing structural disorder in one homodimer species

**DOI:** 10.1074/jbc.RA119.012335

**Published:** 2020-05-13

**Authors:** Cian Stutz, Stanislas Blein

**Affiliations:** Department of Antibody Engineering, Ichnos Sciences S.A., Biopôle Lausanne–Epalinges, Epalinges, Switzerland

**Keywords:** antibody, bispecific engagement by antibodies based on the T-cell receptor (BEAT), CH3, heterodimer, knobs-into-holes (KiH), protein engineering, protein folding, protein stability, protein structure, structural disorder

## Abstract

We previously reported efficient heavy-chain assembly of heterodimeric bispecific antibodies by exchanging the interdomain protein interface of the human IgG1 CH3 dimer with the protein interface of the constant α and β domains of the human T-cell receptor, a technology known as bispecific engagement by antibodies based on the T-cell receptor (BEAT). Efficient heterodimerization in mammalian cell transient transfections was observed, but levels were influenced by the nature of the binding arms, particularly in the Fab-scFv-Fc format. In this study, we report a single amino acid change that significantly and consistently improved the heterodimerization rate of this format (≥95%) by inducing partial disorder in one homodimer species without affecting the heterodimer. Correct folding and assembly of the heterodimer were confirmed by the high-resolution (1.88–1.98 Å) crystal structure presented here. Thermal stability and 1-anilinonaphthalene-8-sulfonic acid–binding experiments, comparing original BEAT, mutated BEAT, and “knobs-into-holes” interfaces, suggested a cooperative assembly process of heavy chains in heterodimers. The observed gain in stability of the interfaces could be classified in the following rank order: mutated BEAT > original BEAT > knobs-into-holes. We therefore propose that the superior cooperativity found in BEAT interfaces is the key driver of their greater performance. Furthermore, we show how the mutated BEAT interface can be exploited for the routine preparation of drug candidates, with minimal risk of homodimer contamination using a single Protein A chromatography step.

Among the myriad of reported bispecific antibody (bsAb) technologies, the heavy chain (Hc) heterodimer format is found in the majority of drug candidates. Favorable physical characteristics, long serum half-life, facile purification, and tunable effector functions are some of the strongpoints of having an Fc-based bsAb format (1, 2). Inherent to Hc heterodimeric bsAbs are the unwanted homodimeric species resulting from the transfection of two different Hcs. Most heterodimerization (HD) technologies incorporate a modified CH3–CH3 interface (Fig. 1) that drives the equilibrium toward the formation of the desired heterodimer. Examples include the knobs-into-holes (KiH) technology (3, 4) and electrostatic steering (5). In addition to driving HD by means of CH3 engineering, heterodimer formation can be influenced at the transfection level: varying the DNA ratios can lead to an equilibrated expression of the two Hcs and thus may increase HD. In our experience, however, adjusting chain ratios is time-consuming and has a limited impact. More preferred is the use of equivalent antibody chain ratios combined with a strong Hc HD technology. Alternatively, different antibody halves may be expressed individually and assembled postproduction (6). Another challenge related to heterodimeric bsAbs is light chain (Lc) mispairing caused by the random pairing of Lcs with Hcs upon co-transfection. This may be circumvented by using a common Lc or, as a more straightforward solution, by the use of single-chain variable fragment (scFv) fusion: variable heavy chain (VH) and variable light chain (VL) domains are genetically fused in one of the Hcs, resulting in the Fab–scFv–Fc format (Fig. 1). Drawbacks of the scFv format include its unpredictable biophysical nature. We found that depending on the characteristics of the scFv, the HD level can be impacted. First, the cause of this may lie in the absence of the CH1 domain that represents an important quality-control checkpoint in the antibody secretion pathway. Lack of regulation may favor scFv-based homodimer formation over heterodimers (7–9). Second, we found that the general expression level of the variable domains also impacts HD when incorporating an scFv in a bsAb. Conceivably, the more efficient the HD interface is, the more it can counteract the aforementioned factors and ensure high HD levels, regardless of the format of the binding arms.

**Figure 1.**
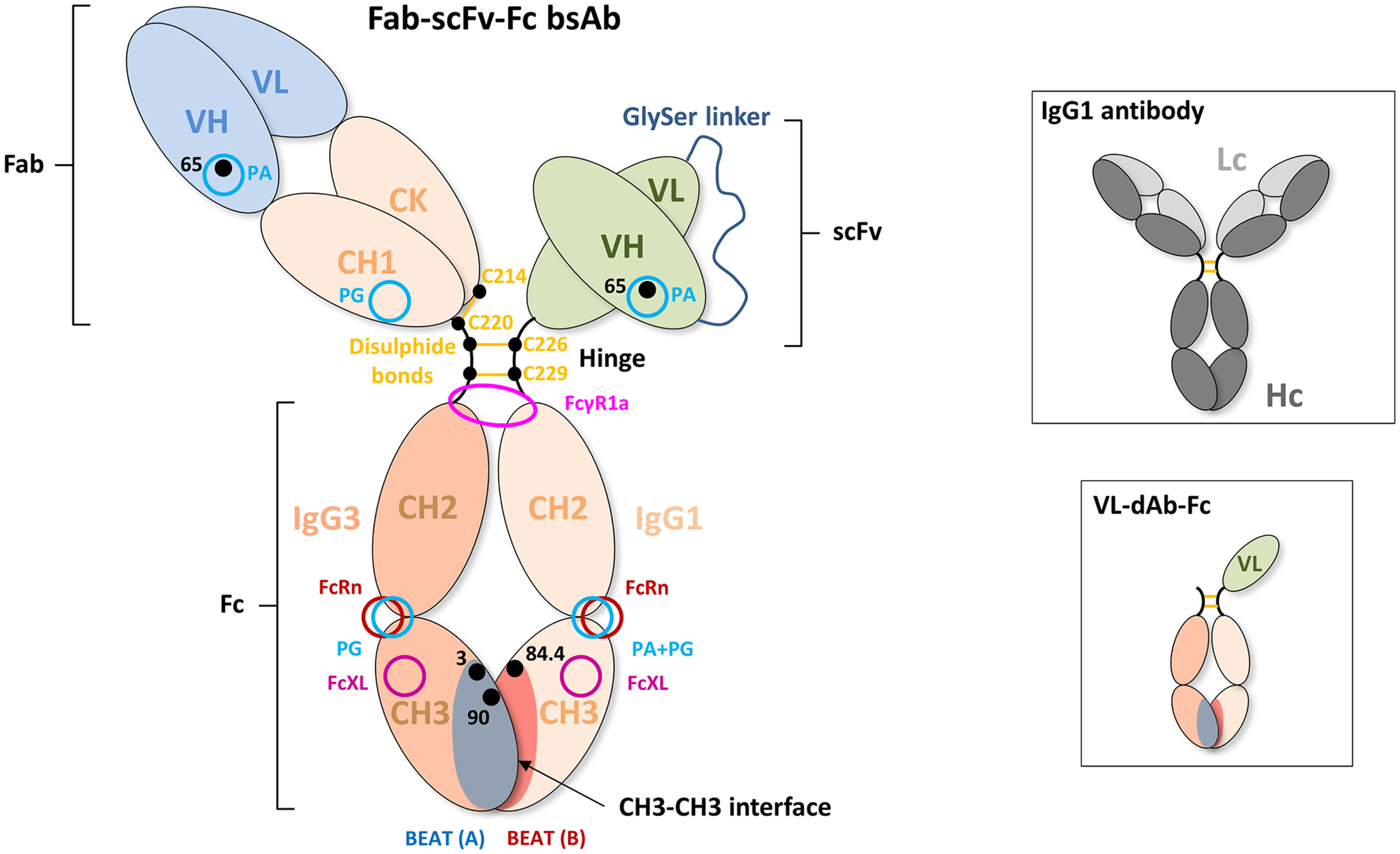
**Schematic drawing of a Fab–scFv–Fc bsAb.** The first Hc noncovalently interacts with the second Hc via the CH3 domains that form the CH3–CH3 interface. Mutations that promote Hc HD, such as those composing the BEAT interface, are generally found in this portion of the antibody. The CH3 domains are connected to the CH2 domains, which together form the antibody Fc portion. The purification resins PA and PG bind at the interface between the CH2 and CH3 domains of the human IgG1 isotype. PA does not bind the IgG3 isotype. The FcRn receptor (neonatal Fc receptor), which promotes long serum half-life, also binds at the CH2–CH3 interface. The CH2 domains are connected to the hinge region. The hinge consists of the lower, middle, and upper hinge, wherein the lower hinge is coded by the CH2 exon. FcγR1a binds asymmetrically across the N-terminal region of the CH2 domains and lower hinge. The middle hinge of the IgG1 isotype contains two cysteine residues (Cys^226^ and Cys^229^) that form intermolecular disulfide bonds with the same cysteines of the second Hc. The N terminus of the upper hinge is connected to the CH1 domain followed by the VH domain. The CH1 domain interacts noncovalently with the constant domain of the Lc, which is termed constant κ (CK) or constant λ (Cλ), depending on the class of Lc. An intermolecular disulfide bond is formed between Hc and Lc, as shown here between Cys^220^ of the upper hinge of the IgG1 isotype and Cys^214^ of the CΚ domain. The VH domain interacts noncovalently with the VL domain. The CH1 domain contains a PG-binding site, and VH domains of the VH3 subclass contain a PA-binding site. PA binding in VH domains of the VH3 subclass can be abrogated using the G65S mutation. To circumvent Lc mispairing, one of the Fab domains can be converted into a scFv by genetic fusion of the VH domain to the VL domain via a flexible linker (generally (Gly_4_-Ser)_3_). The resulting domain is then fused to the hinge region. In the case of the VL–dAb–Fc format (*inset*), a VL-dAb is fused directly to the N terminus of the hinge of one of the Hcs. Cysteine residues are numbered according to the Eu numbering system, VH residues are numbered according to the Kabat numbering system, and CH3 residues are numbered according to IMGT.

We previously reported very efficient HD driven by the BEAT interface, which resulted from grafting the natural human T-cell receptor (TCR) constant domain α–constant domain β heterodimeric interface onto the IgG1 CH3 homodimer domain pair (10). We monitored heterodimer formation in Fc fragments and introduced for this purpose a VL domain antibody (VL-dAb) at the N terminus of one Fc chain to create a difference in molecular mass between homo- and heterodimer species (VL–dAb–Fc format; Fig. 1, *inset*). In this experimental setup, HD rates were consistently above 90–94% in transient HEK transfections. When switching to the Fab–scFv–Fc bsAb format, HD levels were lower and generally at ∼75% in transient transfections. As described above, HD levels depended on the physical attributes of the variable domains used. Thus, we set out to achieve increased HD levels, regardless of the characteristics of the variable domains, by further engineering the BEAT interface. We anticipated that increased HD could be attained either directly by further stabilizing the heterodimeric interface or indirectly by destabilizing the interface in the unwanted homodimeric species.

Using a structure-guided approach and molecular modeling, we identified mutation D84.4Q in the BEAT CH3 (B) domain (according to IMGT numbering or D401Q according to Eu numbering) (11, 12), which restored near perfect HD in the Fab–scFv–Fc format, as previously seen with the original BEAT interface in the VL–dAb–Fc format. With this optimized HD interface, the variable domains used were found to have little impact on HD levels, consistently reaching ≥95%.

Here we report the structural and biophysical characterization of homo- and heterodimers encompassing the D84.4Q substitution *versus* nonmutated controls. We found that D84.4Q induced partial disorder in the Fc of homodimers carrying the mutation, as well as the excessive formation of half-antibodies—two phenomena not observed in D84.4Q-containing heterodimers that were stable and undistinguishable from nonmutated heterodimers as seen in the crystal structure reported herein. We found that D84.4Q homodimers lack Protein A (PA) binding, and we show how this loss of function can be exploited to produce stable bsAbs via a single PA purification step with minimal risk of contaminating species, a key advantage when screening bsAbs for drug discovery.

Finally, our results shed structural insights on bsAb Hc hetero- *versus* homodimer assembly, and the nature of half-antibodies on which only little data could be found prior to this work (13). As in our previous studies (10), antibodies based on KiH were used as comparators. The KiH technology can be considered the industry standard in terms of Hc heterodimerization but has been reported to suffer from variable heterodimer purity (14). Our findings may also be of relevance to technologies where assembly of half-antibodies into bsAbs occurs postproduction.

## Results

### A single mutation increases the heterodimerization level of BEAT Fc by disfavoring homodimer formation

As a starting point for engineering, different Fab–scFv–Fc constructs based on the previously reported BEAT interface were assessed. Fab arms encompassed a VH domain fused to an IgG1 CH1 domain and hinge followed by IgG3 CH2 and CH3 domains having the BEAT (A) substitutions (S20K, T22V, K26T, K79Y, F85.1S, Y86V, K88W, and T90N, with IMGT numbering) and the Q3A mutation previously reported to increase HD in the VL–dAb–Fc format (10). ScFv arms included a VH–VL domain fused to an IgG1 hinge via a Gly_4_-Thr linker followed by a CH2 and CH3 domain having the BEAT (B) substitutions (Q3E, Y5A, L7F, S20T, T22V, K26T, T81D, V84L, D84.2E, F85.1A, Y86S, K88R, and T90R). IgG3 domains, which naturally do not bind PA, were used in BEAT (A) chains to abrogate PA binding thereof (15). Upon co-transfection, the Fab arm, Lc, and scFv arm assembled into bispecific molecules having Hcs of different avidity for PA, thereby allowing separation of homo- and heterodimer species. Importantly, variable domains were selected from different VH subclasses, because the VH3 subclass is known to bind PA (16). The various bsAbs were carefully designed not to encompass VH3-type variable domains on the IgG3-based Hcs to avoid reducing or nullifying the difference in PA avidity between Hcs. Alternatively we used VH3-type domains carrying the G65S substitution (Kabat numbering), a single VH framework mutation that abrogates PA binding (17).

**Figure 2.**
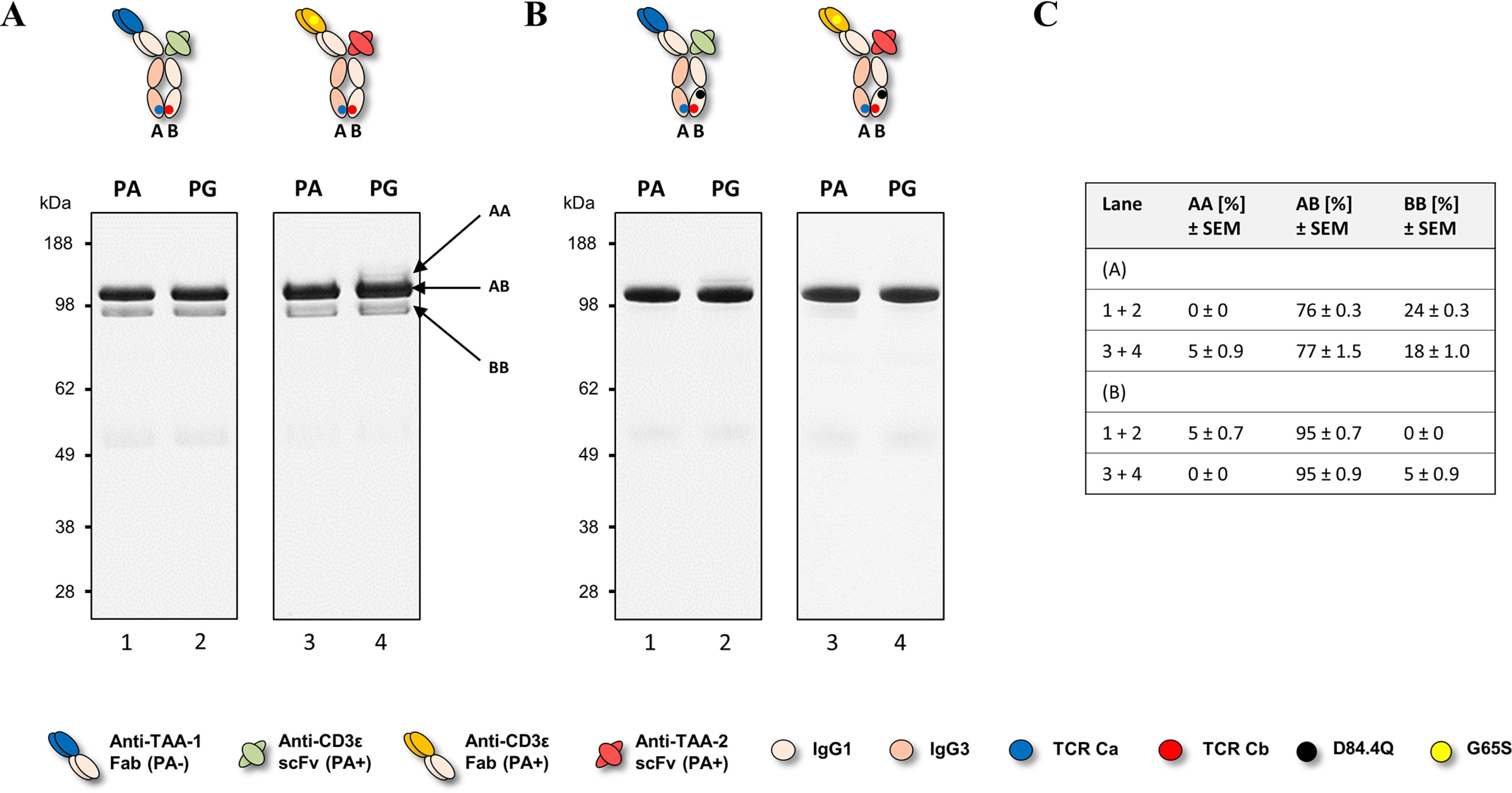
**Non-reduced SDS-PAGE analysis of hetero- and homodimer content of two different BEAT constructs.** bsAbs were transiently expressed in HEK293-EBNA, purified by PA or PG chromatography, and analyzed by SDS-PAGE. *PA*+ indicates the presence of a PA-binding site, and *PA*− indicates the absence of a PA-binding site. For engineering, the G65S mutation was used to abrogate PA binding in VH3-type variable domains when needed. *A*, *lanes 1* and *2*, anti–TAA-1 × anti-CD3ε bsAb. *Lanes 3* and *4*, anti-CD3ε × anti–TAA-2 bsAb. Bands for homodimers (*AA* and *BB*) and heterodimers (*AB*) are annotated with *arrows*. *B*, the same bsAbs as in *A* but carrying the D84.4Q substitution in the CH3 domain of the BEAT (B) chain. *C*, summary of heterodimer content. Percentages were derived from the combined analysis of the PA and PG pulldowns.

**Figure 3.**
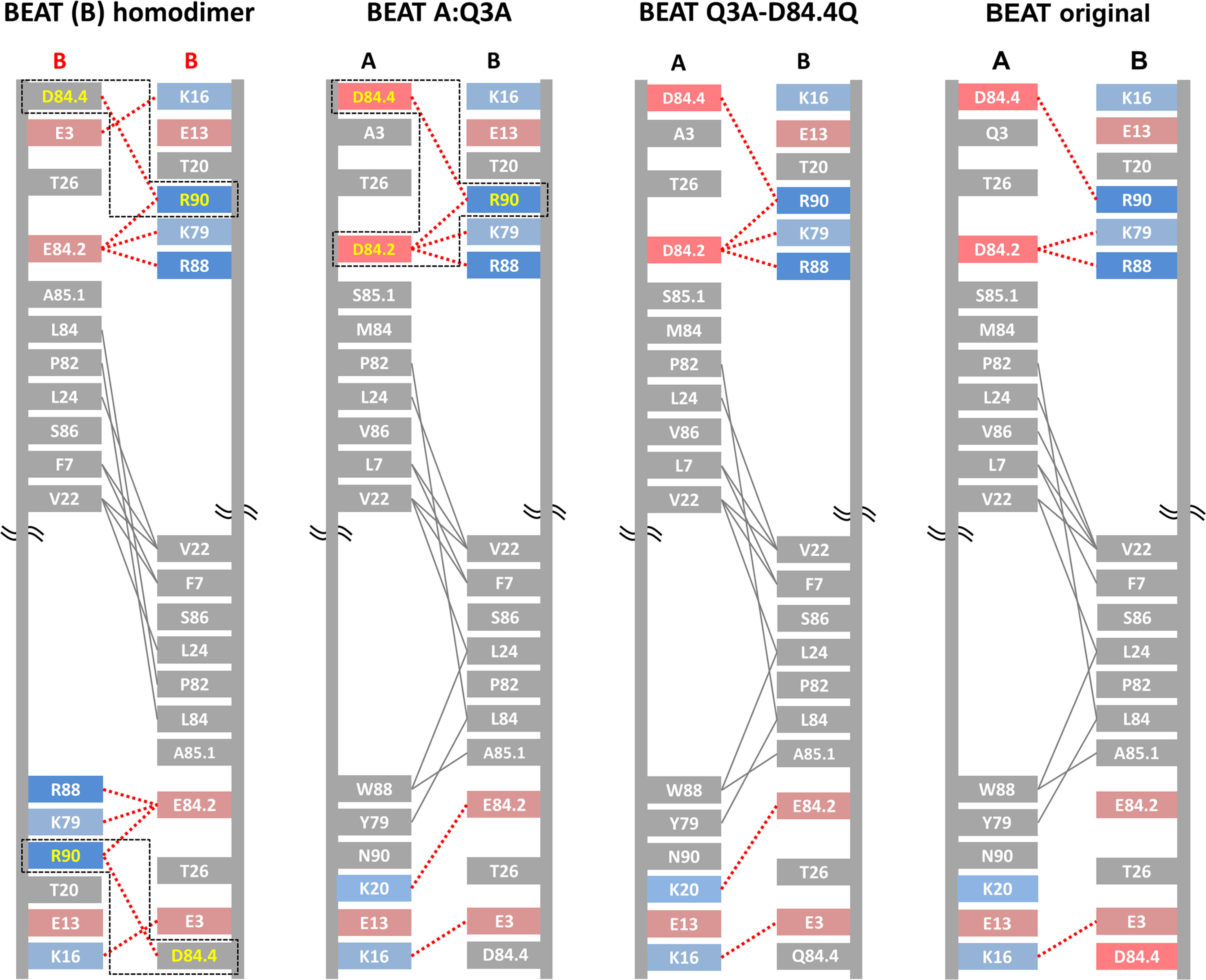
**Schematic diagrams depicting the CH3 interfaces of the BEAT (B) homodimer, the BEAT heterodimer containing the additional Q3A substitution in BEAT (A), the BEAT Q3A-D84.4Q heterodimer, and the original BEAT heterodimer.** Interdomain interactions were calculated from the homology models for the first two and from the crystal structures for the latter two (PDB codes 6G1E and 5M3V, respectively). Homology models were generated based on the crystal structure of the original BEAT Fc. The IMGT numbering is used. Charged residues are colored in *red* (negative) or *blue* (positive). Hydrophobic interactions are in *gray lines*, and electrostatic interactions are in *dashed red lines*. The symmetric electrostatic interactions between Asp^84.4^ and Arg^90^ in the BEAT (B) homodimer as well as the asymmetric interactions between Asp^84.4^, Asp^84.2^, and Arg^90^ in the BEAT Q3A heterodimer are *boxed* in *dashed black lines*, and the residues are colored in *yellow*.

Plasmids carrying individual chains were transfected into HEK cells at a 1:1:1 molar ratio, and supernatants were purified by PA or Protein G (PG), with the latter allowing capture of all species. Homo- and heterodimer formation were assessed by non-reduced SDS-PAGE and quantified by scanning densitometry of the gel bands. Fig. 2*A* shows the distribution of homo- and heterodimeric species for two different bsAb combinations: anti–TAA-1 × anti-CD3ε (Fab arm: tumor-associated antigen 1 (TAA-1) of the VH2 subclass × scFv arm: anti-CD3ε scFv of the VH3 subclass) and anti-CD3ε × anti–TAA-2 (Fab arm: anti-CD3ε of the VH3 subclass abrogated for PA binding via the G65S substitution × scFv arm: anti–TAA-2 of the VH3 subclass). We found that although the percentages of BEAT (A) and BEAT (B) homodimers varied depending on the variable domains used, BEAT (B) homodimers were the most represented contaminant species, an observation that we made also for other variable domain combinations not shown here. Consequently, we speculated that reducing the propensity of BEAT (B) homodimer assembly would shift the equilibrium in favor of heterodimer formation. Based on the crystal structure of the original BEAT interface (BEAT Fc, PDB code 5M3V), we generated a computational model of the BEAT (B) homodimer by homology modeling. The interaction network of the resulting model was analyzed with the aim to identify positions that could destabilize the homodimer (Fig. 3, *first panel* from the *left*). We identified residues Asp^84.4^ and Arg^90^ in BEAT (B) as potential candidates for destabilization of the BEAT (B) homodimer. Asp^84.4^ of the first chain forms an electrostatic interaction with Arg^90^ of the second chain. The same is true for the reciprocal set of residues. We hypothesized that disrupting this symmetric set of electrostatic interactions could destabilize the BEAT (B) homodimer and thereby disfavor its formation. Accordingly, we mutated position Asp^84.4^ in BEAT (B). Analogously to the interface grafting procedure described previously (10), glutamine was chosen based on the amino acid residue found in the TCR constant domain β at the equivalent 3D position when superimposing the BEAT Fc and the TCR constant domain α–constant domain β complex structure (TCR, PDB code 1KGC) (18).

We introduced the D84.4Q substitution in the BEAT (B) chain of the bsAbs described above. The antibodies were produced as before, and homo- and heterodimeric species were assessed. Although the anti–TAA-1 × anti-CD3ε bsAb containing the original interface yielded a HD level of 76%, with BEAT (B) homodimers at 24% and no BEAT (A) homodimers (Fig. 2*A*, *lanes 1* and *2*), the same antibody containing the D84.4Q mutation in BEAT (B) yielded a HD level of 95%, with 5% of BEAT (A) homodimers and no BEAT (B) homodimers (Fig. 2*B*, *lanes 1* and *2*). Similarly, the anti-CD3ε × anti–TAA-2 bsAb with the D84.4Q substitution in BEAT (B) showed an increase in HD from 77% (Fig. 2*A*, *lanes 3* and *4*) to 95% with only 5% BEAT (B) homodimers formed and no detectable BEAT (A) homodimers observed (Fig. 2*B*, *lanes 3* and *4*). The significant increase in HD to levels ≥95% as a result of the D84.4Q mutation in BEAT (B) was also observed for the HER3 × HER2 bsAb previously described (10) and could reliably be observed for other combinations of antigen-binding moieties (data not shown). It is noteworthy at this point that an alternative approach to disrupt the symmetric ionic interactions between Asp^84.4^ and Arg^90^ was back-to-wild-type (WT) mutation R90T in BEAT (B). We previously reported that this led to a small increase in HD levels for the VL–dAb–Fc format (10), but we did not observe a significant improvement in the Fab–scFv–Fc format (data not shown). This may be explained by the fact that although the symmetric ionic interactions with Asp^84.4^ in the BEAT (B) homodimer were disrupted by R90T, the asymmetric interactions made with Asp^84.4^ and Asp^84.2^ in the heterodimer were also disrupted and therefore may have canceled out any improvement in HD (Fig. 3, *first* and *second panel* from the *left*).

### The crystal structure of BEAT Fc Q3A-D84.4Q shows little deviation from the original BEAT Fc structure

**Figure 4.**
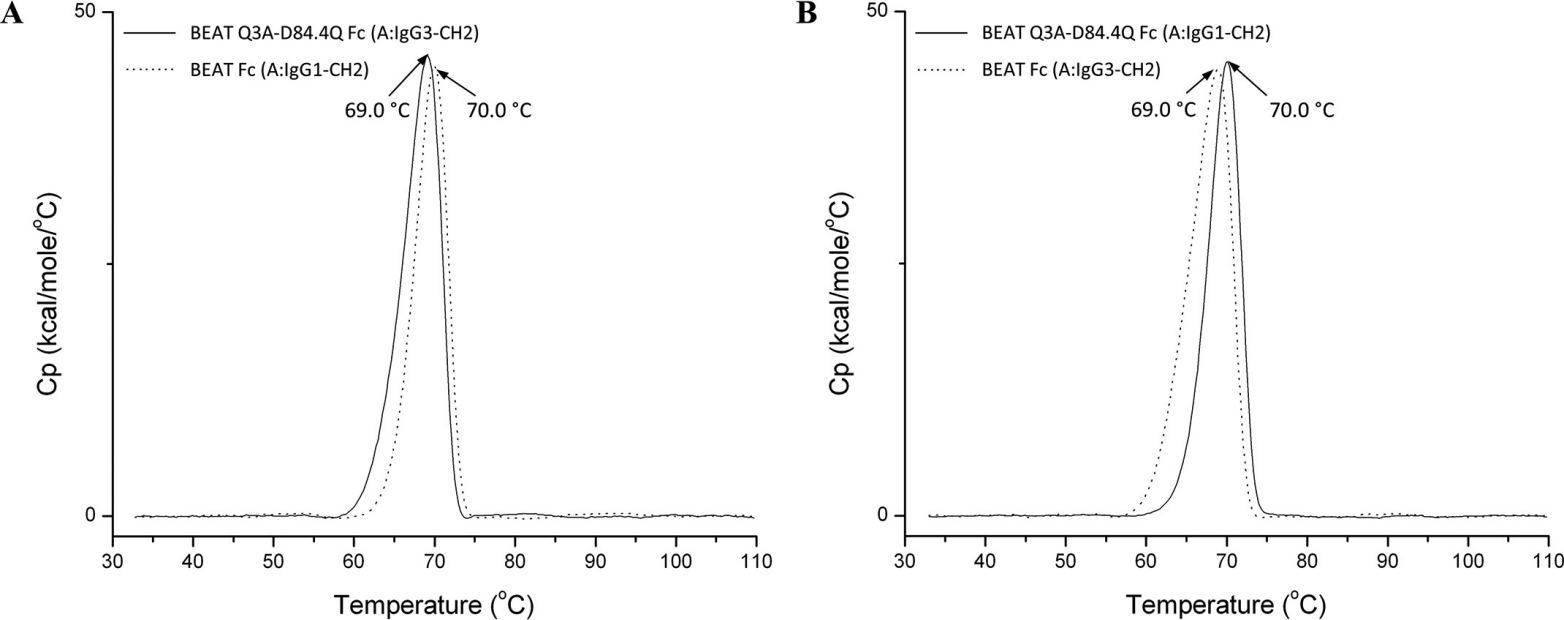
**Thermal stability by DSC showing that D84.4Q has no impact on stability in the heterodimeric context.** *A*, an overlay of BEAT Q3A-D84.4Q Fc (*solid line*) and BEAT Fc (*dashed line*) is shown. The first was built with an IgG3 CH2 domain in the BEAT (A) chain, whereas the latter had an IgG1 CH2 domain. The peak corresponds to the melting transitions (*T*_m_) of both the CH2 and CH3 domain as the transitions overlap. The *T*_m_ values were ∼69.0 and ∼70.0 °C, respectively. *B*, similar to before but BEAT Q3A-D84.4Q Fc (*solid line*) encompassed an IgG1 CH2 domain in the BEAT (A) chain, whereas BEAT Fc (*dashed line*) had an IgG3 CH2 domain. The *T*_m_ values were ∼70.0 and ∼69.0 °C, respectively.

To investigate the molecular basis of HD improvement resulting from the D84.4Q mutation, the X-ray structure of the new BEAT Fc was solved. Chain (A) contained an IgG1 hinge (starting at Asp^221^, with Eu numbering) and an IgG3 CH2 and CH3 domain with the BEAT (A) substitutions, as well as the Q3A mutation in CH3. We previously reported the Q3A mutation to be HD-enhancing but did not include it in the initial construct of which we solved the crystal structure (10). Chain (B) was engineered with an IgG1 hinge, CH2 and CH3 domain with the BEAT (B) substitutions including D84.4Q. We named this construct BEAT Q3A-D84.4Q Fc. The transient expression level in HEK was good and similar to that of the original BEAT Fc. Thermal stability by differential scanning calorimetry (DSC) was reduced by 1 °C to 69 °C compared with BEAT Fc (Fig. 4*A*). By melting the same molecule in which the IgG3 CH2 domain of BEAT (A) was substituted with that of IgG1, we determined that it was the IgG3 CH2 domain that led to the 1 °C loss in thermal stability and not the D84.4Q substitution (Fig. 4*B*).

**Figure 5.**
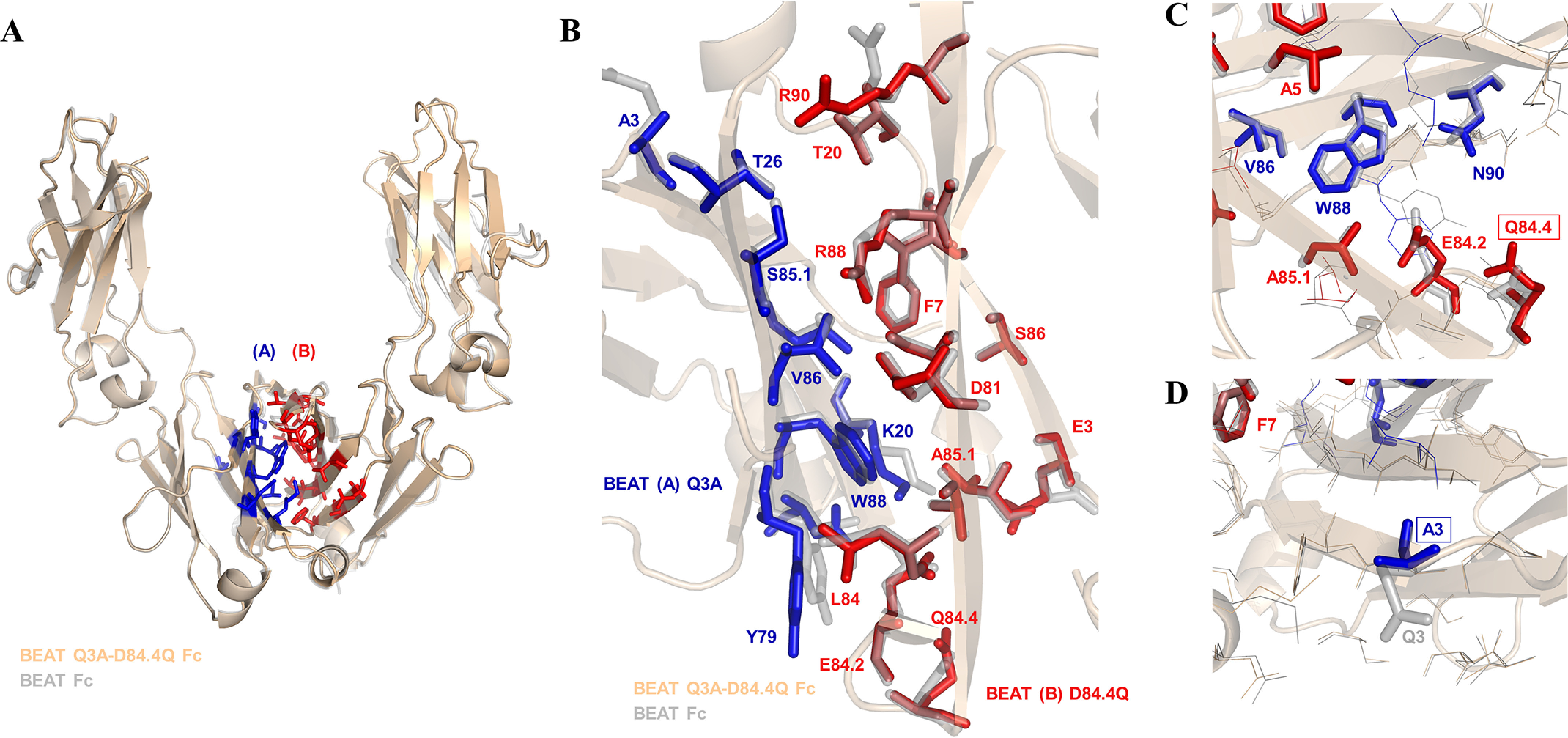
**Crystal structure of the BEAT Q3A-D84.4Q Fc.** *A*, ribbon diagram. Structural alignment of the original BEAT Fc structure (PDB code 5M3V) with that of BEAT Q3A-D84Q Fc (PDB code 6G1E). BEAT substitutions are in *blue* and *red*. *B*, close-up view of the same structural alignment. Side chains of the original BEAT Fc are in *gray*. A significant conservation of side-chain conformations could be observed (IMGT numbering). *C*, close-up showing position Gln^84.4^ and residues in its immediate environment, highlighting that no significant structural changes were induced by the mutation. *D*, close-up showing position A3 and residues in its immediate surroundings.

BEAT Q3A-D84.4Q Fc was crystallized, and its 3D structure was solved at high resolution (Table 1). The same so-called “horseshoe” structure as that seen in any natural IgG antibody was observed (Fig. 5*A*), with little deviation from other experimentally solved structures including the previously reported BEAT Fc structure (root-mean-square deviation of 0.63 Å *versus* PDB code 2WAH and 0.30 Å *versus* PDB code 5M3V when superimposing the CH3 domains on their Cα traces). Analysis of the BEAT substitutions in the engineered CH3 domains revealed identical side-chain orientations for the majority of amino acids (∼87%) compared with the original BEAT Fc (Fig. 5*B*). Interestingly, however, the side chain of position Arg^90^ in BEAT (B) changed its conformation, leading to an additional ionic interaction compared with the original BEAT Fc, potentially contributing to the favorable HD levels (Fig. 3, *third versus fourth panel* from the *left*). Also, Lys^20^ in BEAT (A) forms an ionic interaction that was not observed in the original BEAT Fc. The conformation of the side chain of position Lys^20^ is slightly shifted compared with that in the original structure, which explains the modified interaction network. The difference in conformation, however, is likely an artifact resulting from the poorly defined electron density for the lysine side chain in both crystal structures. A direct link between the change in side-chain conformation of Arg^90^ and the D84.4Q or Q3A mutations could not be derived: the environment around the D84.4Q substitution showed no structural alterations, with side chains (BEAT substitutions and others) keeping the same conformation as in the structure lacking the mutation (Fig. 5*C*). Similarly, substitution Q3A did not induce any structural changes, and the side-chain conformations of positions in its immediate surroundings remained the same (Fig. 5*D*).

**Table 1:** **Data collection and refinement statistics of the BEAT Q3A-D84.4Q Fc crystal structure**

	BEAT Q3A-D84.4Q Fc
**Data collection**	
Wavelength (Å)	0.966
Space group	P2_1_2_1_2_1_
Cell dimensions	
*a*, *b*, *c* (Å)	49.7, 73.0, 140.9
α, β, γ (°)	90.0, 90.0, 90.0
Resolution (Å)*^a^*	30.00–1.88 (1.98–1.88)
*R*_merge_ (%)	0.080 (0.622)
*R*_meas_ (%)	0.090 (0.707)
*R*_pim_ (%)	0.041 (0.332)
*I*/σ*I*	8.5 (2.0)
Completeness (%)	99.5 (99.9)
Redundancy	4.3 (4.3)
**Refinement**	
Resolution (Å)	30.00–1.88 (1.93–1.88)
No. reflections	40,305/2929
*R*_work_/*R*_free_	0.231/0.273
No. atoms	
Protein	3354
Sugar	220
Water	321
B factors	
Protein	56.8
Sugar	83.5
Water	51.0
Root-mean-square deviations	
Bond lengths (Å)	0.007
Bond angles (°)	1.372

*^a^*The values in parentheses are for highest-resolution shell.

### D84.4Q substitution abrogates PA and PG binding in the Fc of the BEAT (B) homodimer species

**Figure 6.**
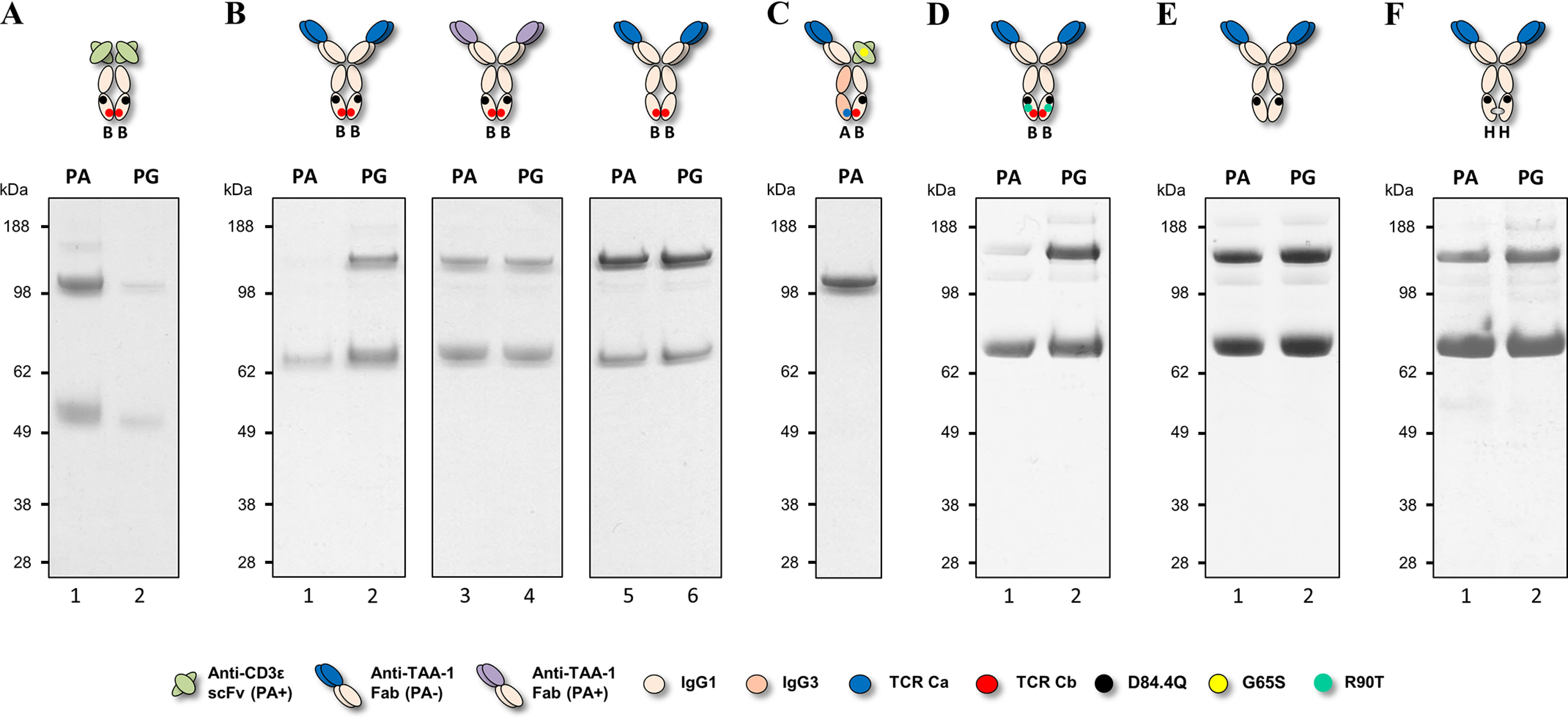
**Non-reduced SDS-PAGE analysis of BEAT (B) homodimers.** Antibodies were transiently expressed, purified by PA or PG chromatography, and analyzed by SDS-PAGE. *A*, anti-CD3ε BEAT (B) D84.4Q homodimer produced for analytical purposes. The *band* just above the 98-kDa molecular mass marker corresponds to the homodimer, and the *band* immediately above the 49-kDa molecular mass marker corresponds to the half-antibody. *B*, *lanes 1* and *2* show the anti–TAA-1 BEAT (B) D84.4Q homodimer wherein the variable domain is of the VH2 subclass. In *lanes 3* and *4*, the VH2-type anti–TAA-1 was replaced with a VH3-type anti–TAA-1. In *lanes 5* and *6*, the D84.4Q substitution was removed in the VH2-type anti–TAA-1 BEAT (B) homodimer. The *upper bands* above 98 kDa correspond to homodimers, and the *bands* above 62 kDa correspond to half-antibodies. *C*, anti–TAA-1 × anti-CD3ε bsAb wherein both variable domains did not bind PA, which confirmed that the PA-binding site in the Fc of the BEAT (B) D84.4Q chain was functional in the context of an heterodimer. *D*, VH2-type anti–TAA-1 BEAT (B) D84.4Q homodimer containing the back-to-WT mutation R90T. *E*, VH2-type anti–TAA-1 homodimeric WT IgG1 antibody with the D84.4Q mutation. *F*, VH2-type anti–TAA-1 KiH (H) homodimer with the D84.4Q mutation.

We succeeded at improving HD of Fab–scFv–Fc BEATs by introducing the D84.4Q substitution that, putatively, destabilized the BEAT (B) homodimer species. For analytical purposes, we produced the BEAT (A) and BEAT (B) D84.4Q homodimers corresponding to the anti–TAA-1 × anti-CD3ε bsAb described above. Upon purification, we found that although BEAT (A) homodimers could readily be purified by PG, BEAT (B) D84.4Q homodimers could only be isolated by PA and did not significantly bind PG (Fig. 6*A*). This suggested, for example, misfolding or conformational changes in the PG-binding site of the BEAT (B) D84.4Q Fc region. Considering that PG binds to almost the same part of the Fc as PA does, *i.e.* the CH2–CH3 interface (19, 20) (Fig. 1), it was surprising to find that the BEAT (B) D84.4Q homodimers bound PA but not PG. We hypothesized that PA binding in the BEAT (B) D84.4Q homodimer was solely mediated by the PA-binding site located in the anti-CD3ε scFv moiety, which encompassed a variable domain of the VH3 subclass and consequently a PA-binding site (16). To assess the contribution of the VH3 variable domain binding to PA, we produced a BEAT (B) D84.4Q homodimer where the anti-CD3ε scFv moiety was replaced by an anti–TAA-1 Fab, which was of the VH2 subfamily and thus did not bind PA. That particular Fab was chosen over an scFv for its good stability and good expression level; in addition the melting transition (*T*_m_) of an scFv in DSC measurements would have overlapped with that of the Fc. We found that, as predicted, this BEAT (B) D84.4Q homodimer no longer bound PA (note that some half-antibodies appeared in the bound fraction, discussed below) (Fig. 6*B*, *lane 1*). PG binding on the other hand was restored (Fig. 6*B*, *lane 2*) because of the secondary PG-binding site located in the CH1 domain (21) (Fig. 1). Replacing the VH2-type anti–TAA-1 Fab with a VH3-type anti–TAA-1 Fab restored PA binding (Fig. 6*B*, *lane 3*). Removing the D84.4Q substitution in a homodimer with the VH2-type anti–TAA-1 Fab restored PA binding (Fig. 6*B*, *lane 5*). Taken together, the PA- and PG-binding sites in the Fc region of a BEAT (B) D84.4Q homodimer are both nonfunctional.

The latter result suggested that the BEAT (B) D84.4Q homodimer Fc portion was misfolded or structurally altered in the PA- and PG-binding regions. However, characterization of numerous BEATs wherein the BEAT (B) CH3 domain contained the D84.4Q substitution revealed no inconsistencies in terms of the structural integrity of the heterodimeric assembly. The functionality of the PA site in the heterodimeric context was further confirmed by studying an anti–TAA-1 × anti-CD3ε bsAb wherein the VH3-type anti-CD3ε scFv carried the G65S mutation (Fig. 6*C*): The BEAT (A) chain did not bind PA (Fab of the VH2 subclass and IgG3-based CH3 domain), and the BEAT (B) D84.4Q chain with the PA-binding site abrogated in the scFv moiety only had its PA-binding site in the Fc region to drive purification, but this was sufficient to readily isolate the heterodimer.

Interestingly, substitution of Arg^90^ in BEAT (B), the residue with which Asp^84.4^ putatively forms an electrostatic interaction in BEAT (B) homodimers (Fig. 3, *first panel* from the *left*), was not required for the PA abrogation phenomenon induced by D84.4Q: a homodimer composed of an anti–TAA-1 Fab and an engineered BEAT (B) chain as described above containing the D84.4Q substitution and the back-to-WT mutation R90T failed to bind PA, whereas PG binding was presumably rescued via the secondary sites in the CH1 domains (Fig. 6*D*). Contrariwise, a similar homodimer with a WT IgG1 CH3 domain, *i.e.* without any BEAT substitutions, containing the D84.4Q mutation was not abrogated for PA binding (Fig. 6*E*). Notably, although PA abrogation was not observed, the mutation in WT IgG1 did induce the formation of significant amounts of half-antibodies (Fig. 6*E*, *band* just above the ∼62-kDa molecular mass marker). Next, we introduced the D84.4Q mutation in a similar homodimer based on the KiH technology (3, 4). The homodimer was engineered as above but carried the “hole” substitutions (denoted H; T366S, L368A, Y407V) in its CH3 domain. KiH (H) was chosen as a recipient for the D84.4Q substitution over KiH “knob” (denoted K; T366W) because we found its characteristics to be the closest match to BEAT (B); both KiH (H) and BEAT (B) are the chains receiving the smaller residues, creating cavities to accommodate the larger residues introduced into KiH (K) and BEAT (A). Furthermore, some characterization data for KiH (H) homodimers had been published (13). Upon purification, we found that this antibody was not abrogated for PA binding (Fig. 6*F*).

### BEAT (B) D84.4Q homodimers show altered biophysical properties beyond their lack of PA and PG binding

**Figure 7.**
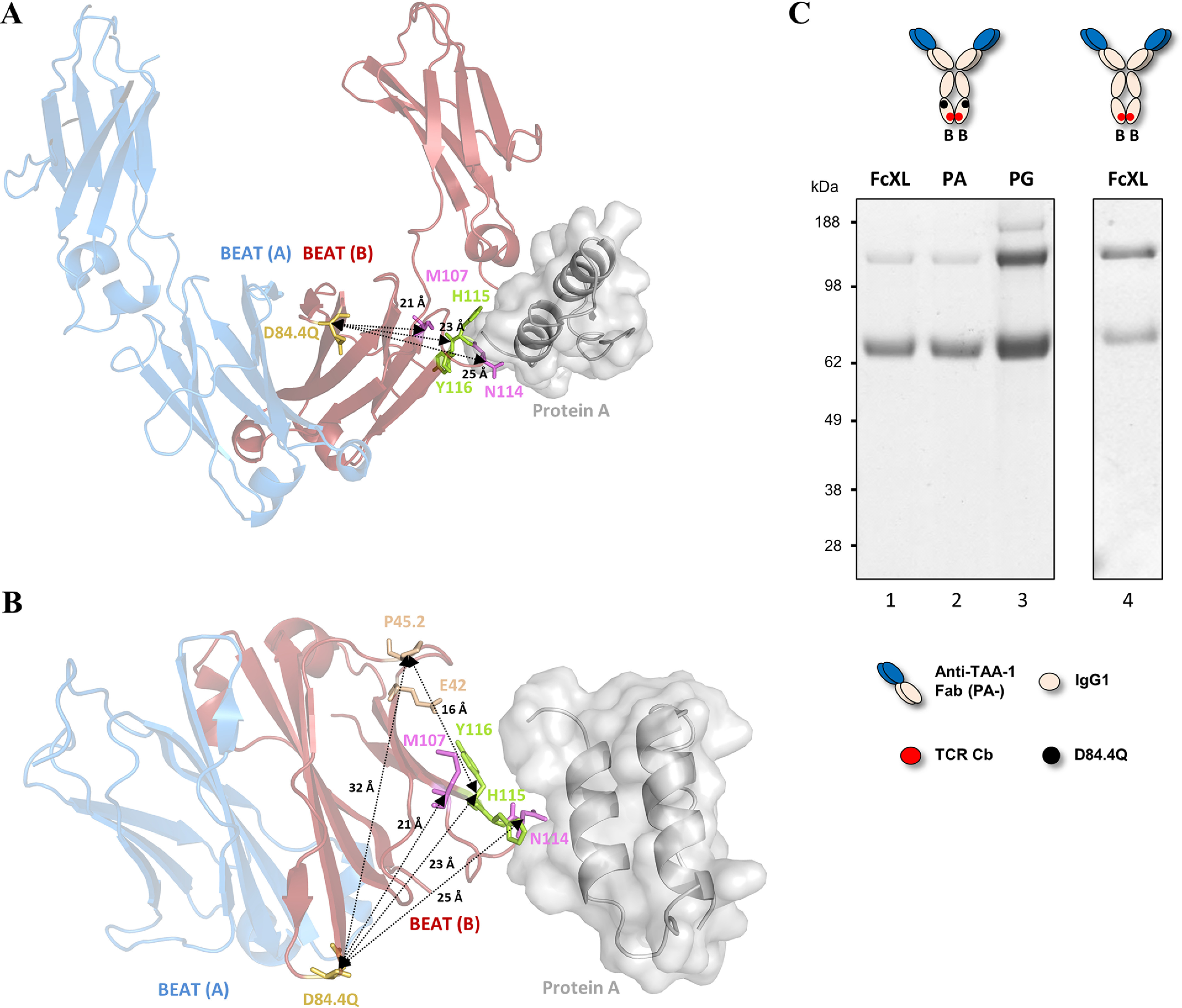
**Molecular details of PA, PG, and FcXL binding sites in the BEAT Fc.** *A*, ribbon diagram of the structure of BEAT Q3A-D84.4Q Fc superimposed with the structure of PA–human Fc complex (PDB code 1FC2; the Fc is not shown). Front view of CH2 and CH3 domains. BEAT (A) is in *blue*, and BEAT (B) in *red*. PA is in *gray*. Position 84.4 is in *yellow*, and residues His^115^ and Tyr^116^ (His^435^ and Tyr^436^, respectively, according to Eu numbering), which make important interactions with PA, are in *green*. PG-binding residues are in *pink*. Distances between Cα of positions 84.4 and Cα 107, 115, and 116 are indicated. *B*, top view of the protein complexes with the CH2 domains hidden. Residues mediating FcXL binding are colored in *wheat*. Distances between Cα atoms are indicated. *C*, BEAT (B) homodimers with and without D84.4Q were transiently expressed, affinity-purified by FcXL, PA, or PG chromatography and analyzed by non-reduced SDS-PAGE. For the BEAT (B) D84.4Q homodimer, purification by PA confirmed the same lack of binding as with the FcXL resin (*lanes 1* and *2*), whereas PG purification confirmed expression of the homodimer (*lane 3*). *Lane 4* shows that a BEAT (B) homodimer lacking the D84.4Q substitution was able to bind the FcXL resin.

Position 84.4 itself is not part of either the PA- or the PG-binding site but is located opposite at the CH3–CH3 interface with distances of ∼23 and ∼21 Å from the closest PA- and PG-interacting residues, respectively (Fig. 7, *A* and *B*) (19, 20). Because there are no direct interactions, only the induction of long-range conformational changes in the Fc of BEAT (B) D84.4Q homodimers by the substitution could explain the lack of binding to both resins.

In an attempt to probe the depth of the structural alterations in the CH3 domains of the BEAT (B) D84.4Q homodimer, the homodimer was purified using FcXL, an affinity matrix that binds to residues Glu^42^ and Pro^45.2^ of the CH3 domain (IMGT numbering) (22). These residues are located at a distance of ∼16 Å from the residues of the PA-binding site and ∼32 Å from position 84.4 (Fig. 7*B*). Intriguingly, the BEAT (B) homodimer containing the D84.4Q substitution did not bind the FcXL resin, revealing that the structural changes are not restricted to the PA- and PG-binding sites but are of a longer range (Fig. 7*C*).

**Figure 8.**
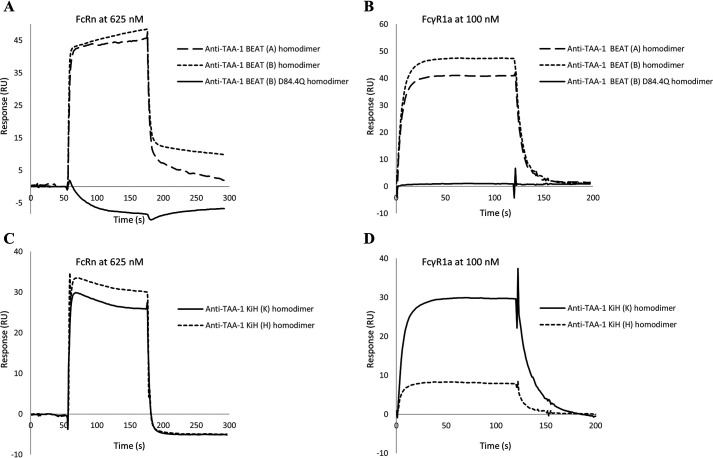
**SPR analysis of FcRn and FcγR1a binding to anti–TAA-1 BEAT and KiH homodimers.** *A*, FcRn injected at 625 nm onto a sensor chip covalently coupled with BEAT (A), BEAT (B), or BEAT (B) D84.4Q homodimers. *B*, FcγR1a injected at 100 nm onto a PA-coupled sensor chip wherein BEAT homo- or heterodimers were previously captured via their VH3-type variable domains. *C*, FcRn injected at 625 nm onto a sensor chip covalently coupled with KiH homo- or heterodimers. *D*, FcγR1a injected at 100 nm onto a PA-coupled sensor chip where KiH homo- or heterodimers were previously captured via their VH3-type variable domains.

Thenceforth, we set out to probe the CH2 domain for its integrity. No affinity resin specific for binding to the CH2 domain could be identified, and thus we decided to examine binding of the Fc receptors (FcR), more specifically the human neonatal Fc receptor (FcRn) and human FcγR1a binding. FcRn binds at the interface between the CH2 and the CH3 domain (Fig. 1), and structural changes induced by the D84.4Q mutation were expected to be reflected in the binding affinity (23). FcγR1a binds asymmetrically across the N-terminal region of the CH2 domains and the lower hinge (Fig. 1) (24, 25). Affinity measurements were performed by surface plasmon resonance (SPR). Antibodies used were of high purity (≥95%), and preparative size-exclusion chromatography (SEC) was used to remove half-antibodies when needed (discussed below). BEAT (B) homodimers had an average binding affinity constant (*K_D_*) of 440 nm for FcRn binding (Table S1), an affinity that is in-line with values reported for human IgG1 (26). Intriguingly, BEAT (B) D84.4Q homodimers did not bind FcRn at all, further supporting the idea of structural changes in the Fc (Fig. 8*A*). Subsequently, we tested FcγR1a binding. We found that although the receptor was readily binding to BEAT (B) homodimers, there was no binding to BEAT (B) D84.4Q homodimers (Fig. 8*B*).

Next, we investigated homodimers based on KiH, with the goal of determining whether alteration in FcR binding is a unique feature of BEAT (B) D84.4Q homodimers. Antibodies were engineered as above, but containing the “knob” and “hole” substitutions (as above) instead of the BEAT substitutions. FcRn binding was assessed (Fig. 8*C* and Table S1). KiH (K) homodimers and KiH heterodimers had *K_D_* values in the expected range. KiH (H) homodimers bound FcRn, although with a 2-fold reduction in affinity. All KiH molecules bound FcγR1a, although the KiH (H) homodimer showed reduced binding (Fig. 8*D*).

### BEAT (B) D84.4Q half-antibodies retain the ability to bind PA

**Figure 9.**
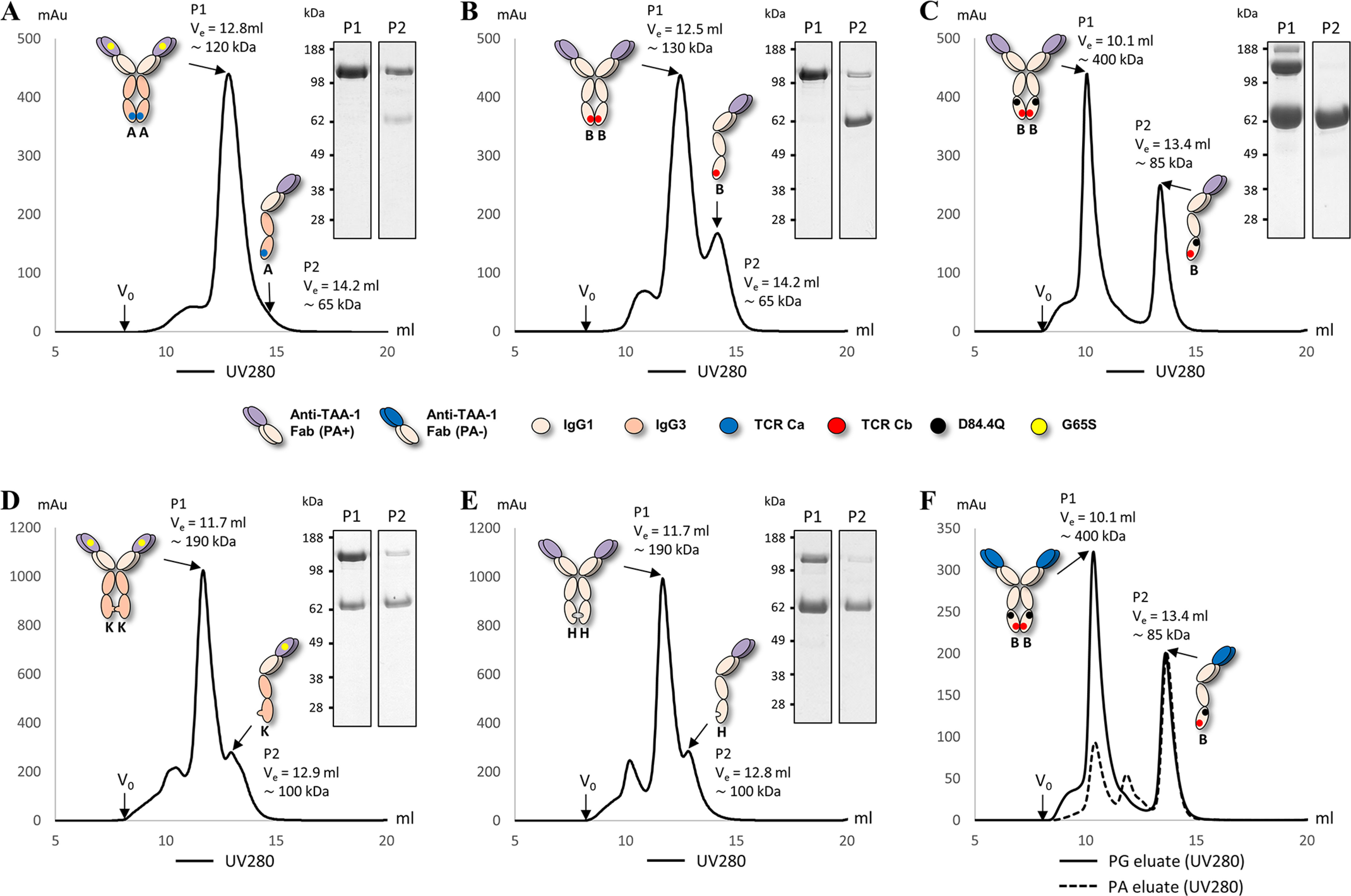
**SEC traces of BEAT and KiH homodimers with non-reduced SDS-PAGE analysis of peak fractions.** *V*_e_ is the volume of elution, and *V*_0_ is the void volume. Peak 1 is denoted *P1*, and peak 2 is denoted *P2*. Molecular mass calculated from a calibration run as indicated. *A*, BEAT (A) homodimer. P2 appeared merely as a shoulder because of the low abundancy of half-antibodies. *B*, BEAT (B) homodimer. *C*, BEAT (B) D84.4Q homodimer. *D*, KiH (K) homodimer. *E*, KiH (H) homodimer. *F*, BEAT (B) D84.4Q homodimer from PA (*dashed line*) and PG (*solid line*) eluates. Both chromatograms are shown overlaid.

Upon purification of BEAT (A) and BEAT (B) homodimers by affinity chromatography, we commonly observed significant amounts of half-antibodies co-eluting with the fully assembled, *i.e.* disulfide-bonded homodimer (Fig. 6*B*, *band* just above the ∼62-kDa molecular mass marker). Such half-antibodies were generally not observed in significant amounts for heterodimeric BEAT bsAbs. Therefore, we attributed this behavior to the poor pairing capabilities of the homodimeric interfaces, which was undoubtedly the engineering objective in the first place. We anticipated that half-antibodies would interfere with the further characterization of the homodimers and thus set out to obtain homodimers free of such contaminants (Fig. 9). As before, we did not use the scFv configuration because of the less favorable properties of this format. The BEAT (A) homodimer was designed with the IgG3 isotype, the VH3 subclass, and the aforementioned G65S substitution to match our original bsAb architecture. Thus, the BEAT (A) homodimer would not bind PA and was therefore purified by PG. BEAT (B) homodimers were designed with the IgG1 isotype and the VH3 subclass that enabled PA purification via the Fc and/or the VH domains depending on the absence or presence of the D84.4Q substitution. The considerable difference in molecular mass between a fully assembled homodimer (145.6 kDa) *versus* a half-antibody (72.8 kDa) suggested preparative SEC as a suitable method for separation. PG eluates of BEAT (A) homodimers generally contained low amounts of contaminating half-antibodies that could readily be separated (Fig. 9*A*). The PA eluate of the BEAT (B) homodimer contained significantly more half-antibodies, but SEC was also successful at isolating this species (Fig. 9*B*). Volumes of elution (*V*_e_) recorded for the first peak (P1) containing the homodimers and the second peak (P2) containing the half-antibodies were in the same range for both BEAT (A) and BEAT (B) homodimers and were found at the expected molecular mass as determined by a calibration run. P2 of BEAT (A) appeared merely as a shoulder because there were not many half-antibodies to begin with. The PA eluate of the BEAT (B) D84.4Q homodimer also separated into two peaks, but P1 showed a much higher molecular mass than expected, reaching ∼400 kDa, whereas the half-antibodies eluted near their expected molecular mass (P2) (Fig. 9*C*). SDS-PAGE revealed that P2 indeed contained half-antibodies, but unexpectedly, P1 also contained significant amounts of these. Initially we hypothesized that the half-antibodies found in P1 were the result of tailing from P2, although near baseline separation of P1 and P2 could be observed. However, a second SEC experiment with the P1 fraction used as input did not further separate half-antibodies from the homodimers (data not shown). This result implied that what we observed in P1 were not in fact half-antibodies. Instead, we postulated that the BEAT (B) D84.4Q homodimers in P1 were composed of a mixture of two species: disulfide-bonded and non–disulfide-bonded in the hinge region (Fig. 1), the latter assembling only via the CH3 domains. Thus, the non–disulfide-bonded species appeared like half-antibodies by SDS-PAGE under nonreducing conditions but co-eluted with the disulfide-bonded homodimers by SEC. Interestingly, when we purified homodimers based on KiH (K) and (H) by SEC, we also found significant amounts of what looked like half-antibodies in the SDS-PAGE analyses of the main peaks for both (Fig. 9, *D* and *E*). However, in contrast to BEAT (B) D84.4Q homodimers, the main peaks were within the expected molecular mass range. For KiH, non–disulfide-bonded homodimers have been previously described (27, 28).

Retrospectively, we analyzed by SEC the PA eluate of the homodimer described in Fig. 6*B* (*lane 1*) in an effort to determine whether the species at ∼62 kDa that retained PA binding contained non–disulfide-bonded homodimers or half-antibodies or both. We found that the PA eluate consisted mostly of half-antibodies (P2) (Fig. 9*F*). Trace amounts of homodimers (P1) resulted from strongly concentrating half-antibodies before injection on the SEC column; we found this could force the pairing of halves into non–disulfide-bonded homodimers after PA purification (data not shown). The PG eluate was run as a control and contained homodimers and half-antibodies; this was anticipated because PG capture occurred via the CH1 domains.

### BEAT (B) D84.4Q homodimers show increased ANS binding

In an attempt to resolve the structural alterations induced by the D84.4Q substitution, we assessed BEAT (B) D84.4Q homodimers in a 1-anilinonaphthalene-8-sulfonic acid (ANS) fluorescence experiment. The binding of the fluorophore ANS is a well-established and sensitive test to probe partial folding in globular proteins. Binding of ANS to a protein induces an increase in its fluorescence at 470 nm upon excitation at 370 nm. ANS binds to solvent-accessible hydrophobic regions in proteins. Although ANS has been shown to bind to surface-exposed patches of nonpolar groups in compactly folded proteins, binding to a partially folded state is much stronger in general compared with the native or fully denatured state (29).

**Figure 10.**
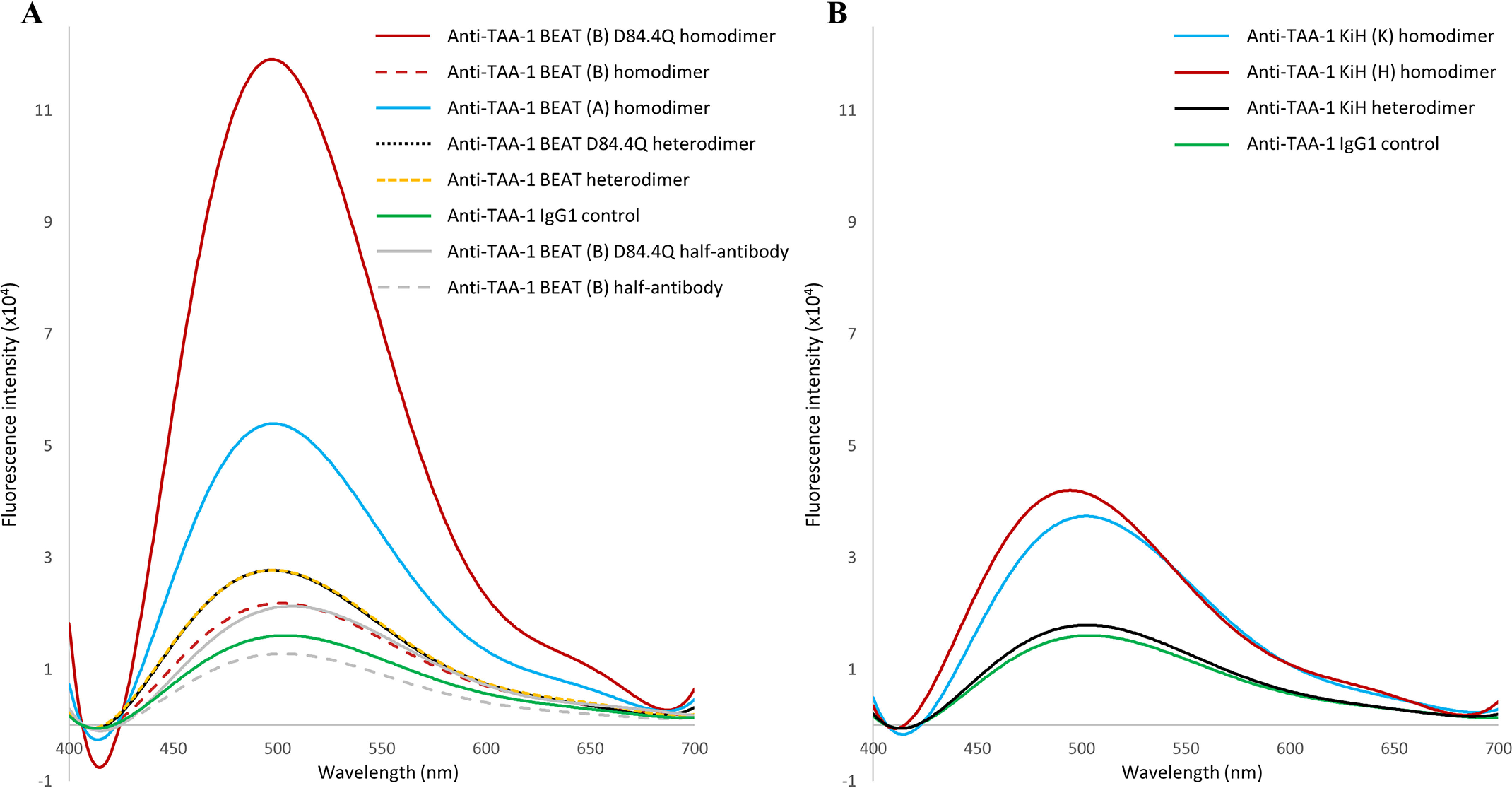
**Fluorescence emission of ANS.** The samples (3.5 μm) were incubated for 20 min with ANS (200 μm) in PBS (pH 7.4) at 20 °C. Excitation was at 370 nm, and emission was measured between 400 and 700 nm. *A*, BEAT antibodies. BEAT (B) D84.4Q homodimers showed the strongest increase in ANS fluorescence over any other molecule tested. *B*, KiH antibodies.

BEAT (A) and (B) homodimers described above were analyzed. Additionally, we produced two anti–TAA-1 Fab × Fab heterodimeric BEAT bsAbs (anti–TAA-1 of the VH3 subclass with the G65S mutation and the IgG3 isotype in BEAT (A), and nonmutated anti–TAA-1 of the VH3 subclass with the IgG1 isotype in BEAT (B)), one with and another one without the D84.4Q substitution in BEAT (B). The WT IgG1 version of the same anti–TAA-1 antibody was used as a control. All antibodies were of high purity. The IgG1 control antibody, the heterodimeric antibodies with and without D84.4Q, as well as BEAT (B) homodimers showed no significant fluorescence emission (Fig. 10*A*). BEAT (A) homodimers showed some fluorescence emission, but the BEAT (B) homodimer containing the D84.4Q substitution showed a far greater fluorescence emission (6-fold compared with the original BEAT (B) homodimer) over any of the tested molecules, indicating that ANS bound to disordered regions. Purified BEAT (B) half-antibodies with and without D84.4Q obtained from the preparative SEC experiments described earlier were also tested, and both showed no increase in fluorescence. Fluorescence experiments were run using different concentrations of ANS. Intensities of fluorescence increased as a function of ANS concentration; however, these scaled consistently among the antibodies tested, and the fold differences remained the same (data not shown).

For comparison, hetero- and homodimers based on KiH were tested for ANS binding (Fig. 10*B*). No significant difference in fluorescence emission between the KiH heterodimer, BEAT heterodimer, and IgG1 control could be observed (Fig. 10, *B versus A*). For the KiH homodimers, fluorescence emission was elevated over IgG1 control and BEAT (B) homodimers, but lower than BEAT (A) homodimers, and well below BEAT (B) D84.4Q homodimers.

### BEAT (B) D84.4Q homodimers have reduced thermal stability

**Figure 11.**
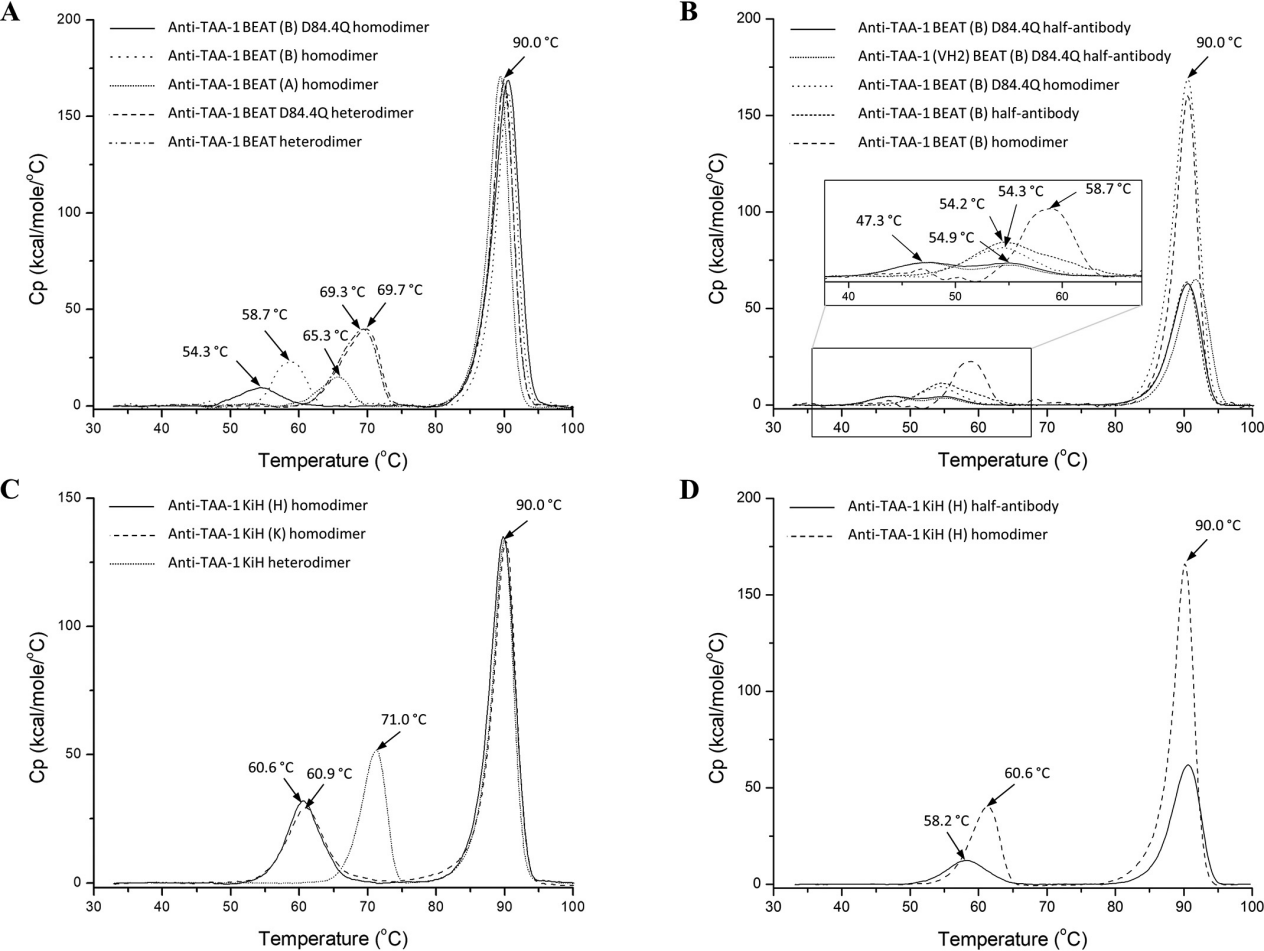
**Thermal stability by DSC, overlay of melting curves.** *A*, an overlay of anti–TAA-1 BEAT bsAbs with and without D84.4Q, as well as the corresponding homodimers. The first transition corresponds to the melting of both the CH2 and CH3 domains, as the peaks overlap. *B*, an overlay of anti–TAA-1 BEAT (B) half-antibodies with and without the D84.4Q mutation. The corresponding homodimers are also shown for comparison. Because of the low Cp values of the transitions for the Fc region, a magnification of the *boxed area* is shown. *C*, an overlay of anti–TAA-1 KiH bsAb and the corresponding homodimers. *D*, anti–TAA-1 KiH (H) half-antibody overlaid with the corresponding homodimer. Each *curve* shows a transition at ∼90 °C that corresponds to the melting of the Fab portion.

Next we investigated the effect of structural alteration on thermal stability. The SEC-purified homodimers described above were assessed by DSC. The same anti–TAA-1 (Fab × Fab format) BEAT heterodimers with and without D84.4Q used in the ANS experiment were included in the analysis. The *T*_m_ for the anti–TAA-1 Fab (∼90 °C) was identical for all molecules tested, indicating that the nature of the Fc region did not impact Fab stability (Fig. 11*A*). In all homo- and heterodimers, the *T*_m_ for the CH2 and CH3 domains overlapped. For the BEAT heterodimers, the *T*_m_ for the Fc (CH2–CH3) domains were identical at ∼69.5 °C, regardless of the D84.4Q substitution, as previously observed (Fig. 4). The *T*_m_ of the BEAT (A) homodimer Fc exhibited a reduced value at ∼65 °C. It is important to note the significant reduction (3-fold) in calorimetric enthalpy (Δ*H*_cal_, which corresponds to the area under the curve) compared with the heterodimeric Fc (Table 2). The BEAT (B) homodimer Fc had a further reduced *T*_m_ value at ∼59 °C as well as a 2-fold lower Δ*H*_cal_ compared with the heterodimeric Fc. Finally, BEAT (B) D84.4Q homodimers had an even further reduced *T*_m_ value for the Fc at ∼54 °C along with a 4-fold reduced Δ*H*_cal_ compared with the heterodimeric Fc. Notably, although we observed two species by SDS-PAGE for the SEC peak 1 fraction (Fig. 9*C*, *P1*), a single thermal transition was observed for the Fc. Purified BEAT (B) D84.4Q half-antibodies were also assessed by DSC and showed a thermal transition at ∼47 °C as well as another at ∼55 °C (Fig. 11*B*). This result suggested the presence of two different species having different residual folding, for example one that binds PA in the Fc and another that does not, which seemed plausible as we purified the half-antibodies via the Fc and the Fab (anti–TAA-1 of the VH3 subclass). Accordingly, we expressed and purified BEAT (B) D84.4Q half-antibodies with the anti–TAA-1 of the VH2 subclass, which was purified by PA binding to the Fc only. In DSC experiments, however, we found that both thermal transitions were still present (Fig. 11*B*). Notably, Δ*H*_cal_ of the homodimeric BEAT (B) D84.4Q Fc was not significantly increased (1.15-fold) compared with the combined Δ*H*_cal_ of the two peaks observed for the BEAT (B) D84.4Q half-antibody Fc (Table 2). Original BEAT (B) half-antibodies on the other hand showed a single *T*_m_ at ∼54 °C and significantly lower Δ*H*_cal_ for the Fc compared with that of the respective homodimeric Fc (1.6-fold). We also tested KiH (K) and (H) homodimers and the corresponding heterodimer under the same experimental conditions (Fig. 11*C*). *T*_m_ values for the homodimeric Fc domains were at ∼61 °C for both. The Fc in the heterodimer had a *T*_m_ of 71 °C and merely a 1.15-fold increase in Δ*H*_cal_ compared with the two homodimers (Table 2). KiH (H) half-antibodies showed a *T*_m_ of ∼58 °C (Fig. 11*D*). Similar to the original BEAT (B), the KiH (H) Fc showed increased Δ*H*_cal_ upon assembly of half-antibodies into homodimers (2-fold) (Table 2).

**Table 2:** **Summary of calorimetric enthalpies (Δ*H*_cal_) and *T*_m_ values of Fc regions. The table presents a summary of calorimetric enthalpies (Δ*H*_cal_) and *T*_m_ values observed for the Fc regions of anti–TAA-1 BEAT D84.4Q, BEAT, and KiH. The data obtained for homodimers, heterodimers, and half-antibodies are shown**

Construct	Δ*H*_cal_ ± S.E.	*T*_m_
	*kcal/mol*	°C
Anti–TAA-1 BEAT (B) D84.4Q half-antibody	5.8 ± 0.6	47.3 and 54.9
Anti–TAA-1 (VH2) BEAT (B) D84.4Q half-antibody	5.1 ± 0.5	47.1 and 55.1
Anti–TAA-1 BEAT (B) half-antibody	8.2 ± 1.5	54.2
Anti–TAA-1 KiH (H) half-antibody	10.4 ± 0.4	58.2
Anti–TAA-1 BEAT (A) homodimer	8.3 ± 0.6	65.3
Anti–TAA-1 BEAT (B) D84.4Q homodimer	6.7 ± 0.8	54.3
Anti–TAA-1 BEAT (B) homodimer	13.2 ± 0.7	58.7
Anti–TAA-1 KiH (K) homodimer	20.9 ± 0.7	60.9
Anti–TAA-1 KiH (H) homodimer	20.5 ± 0.5	60.6
Anti–TAA-1 BEAT D84.4Q heterodimer	27.0 ± 0.7	69.7
Anti–TAA-1 BEAT heterodimer	26.9 ± 0.7	69.3
Anti–TAA-1 KiH heterodimer	24.0 ± 0.6	71.0

## Discussion

Using a structure-guided design approach, we engineered our original BEAT interface to increase HD levels in the Fab–scFv–Fc format under transient expression conditions. We found that mutation D84.4Q in the BEAT (B) CH3 domain significantly and consistently increased HD, whereas the formation of BEAT (B) homodimers was almost completely abolished (≤5%; Fig. 2). Upon solving the crystal structure of the mutant, we found that both the original and mutated BEAT Fc fragments were undistinguishable (Fig. 5). Surprisingly, however, we discovered that BEAT (B) homodimers carrying the D84.4Q mutation no longer bound PA and PG in the Fc region (Fig. 6, *A* and *B*). However, within the corresponding heterodimer, the BEAT (B) D84.4Q chain did bind PA through the Fc (Fig. 6*C*). It is important to note that although the D84.4Q mutation abrogated PA binding via the Fc, homodimers could still bind through their variable domains if they were of the VH3 subclass, thereby enabling the assessment of the HD levels mentioned above (Fig. 2). Interestingly, implementing the D84.4Q substitution in the natural IgG1 Fc or a KiH heterodimeric Fc did not lead to a loss of PA or PG binding (Fig. 6, *E* and *F*). Thus, it was the specific combination of this mutation with the BEAT interface substitutions that led to the phenomena. Notably, the D84.4Q mutation is located at a significant distance from the PA/PG-binding area, which suggested that the observed lack of binding was not the result of directly disrupting interactions between residues from the Fc region and PA or PG. Rather we hypothesized that, unexpectedly, D84.4Q led to long range conformational changes in the homodimer Fc as a result of poorly compatible pairing of the CH3 domains. D84.4Q-containing homodimers no longer bound to FcRn, FcγR1a, and the anti-CH3 affinity matrix FcXL (Figs. 7*C* and 8, *A* and *B*), findings that supported larger conformational changes. Conversely, in the case of the heterodimer, the CH3 domains perfectly complemented each other, leading to proper assembly and functional binding sites in the Fc.

The unexpected phenomena induced by the D84.4Q mutation led us to study Hc homodimers and their half-antibody counterparts in more detail. The latter caught our interest because of the significant proportion of half-antibodies in homodimer preparations; heterodimer preparations generally only showed trace amounts of half-antibodies (≤1%). SEC separation of homodimers from halves revealed that BEAT (B) D84.4Q homodimers had a strongly increased hydrodynamic radius (Rh) over the original BEAT (B) homodimers (Fig. 9). Conceivably, the D84.4Q mutation induced structural changes such as unfolding in the Fc, reducing the retention time in the gel matrix. As anticipated, SEC allowed separation of half-antibodies from homodimers, and notably, half-antibodies migrated at the expected molecular mass (Fig. 9*C*). SEC also revealed another species, which appeared to be half-antibodies by SDS-PAGE that co-eluted with the homodimers. This could not be observed for the original BEAT (B) homodimers. For comparison, KiH homodimers were prepared and assessed by SEC. Studies of KiH have reported covalent and noncovalent forms of KiH homodimers in which the noncovalent form lacks the disulfide bonds in the hinge but antibody halves are held together by interactions in the Fc (27, 28). Noncovalent homodimers would be expected to migrate at the same *V*_e_ as covalent homodimers by SEC but in nonreducing SDS-PAGE would disassemble into halves. Accordingly, SEC of KiH homodimers revealed significant amounts of a species in the homodimer peak that migrated like half-antibodies by SDS-PAGE (Fig. 9, *D* and *E*). The increase in Rh was less significant than for the BEAT (B) D84.4Q homodimers. Zhang *et al.* (13) attributed the increase in Rh of the KiH (H) homodimer to a reduced compactness of the domains caused by their poor pairing capabilities. This also may explain the relatively mild reduction in FcRn and FcγR1a binding compared with the corresponding KiH heterodimer (Fig. 8, *C* and *D*). Complete abrogation of FcRn and FcγR1a binding, however, appeared to be a unique property of the BEAT (B) D84.4Q homodimer. Thus, as opposed to the BEAT (B) D84.4Q homodimers, the Fc in KiH homodimers appeared to be structurally intact, yet a similar phenomenon concerning the co-migrating species by SEC was most likely occurring in BEAT (B) D84.4Q homodimer preparations: the SEC fraction of homodimers appearing as half-antibodies by SDS-PAGE was likely a non–disulfide-bonded form (in the hinge) that, despite the altered structural integrity of the Fc, retained the ability to dimerize via the CH3–CH3 interface. Interestingly, the D84.4Q mutation differently impacted homodimers and half-antibodies: half-antibodies appeared to be better folded given that they retained the ability to bind PA in the Fc and had expected Rh by SEC (Figs. 6*B*, *lane 1*, and 9*C*). It is conceivable that disorder arises only upon assembly of half-antibodies into homodimers, with or without hinge disulfide bonding. When we produced D84.4Q-containing heterodimers, we did not observe significant amounts of half-antibodies or non–disulfide-bonded assemblies, suggesting that the half-antibodies were quickly assembled into the more favorable heterodimeric assembly having full structural integrity (Fig. 6*C*).

The concept of disorder in the Fc region is further supported by ANS data where BEAT (B) D84.4Q homodimers showed a 6-fold increase of fluorescence emission over control molecules (Fig. 10*A*). Conversely, the fraction of half-antibodies separated by SEC showed no significant ANS fluorescence, which is in line with the idea of a higher degree of folding for this species and its ability to bind PA. KiH homodimers showed only a minimal increase in ANS fluorescence over the corresponding heterodimer (∼2-fold), far from the degree observed for BEAT (B) D84.4Q homodimers (Fig. 10*B*). The mild increase in ANS fluorescence for KiH could be explained by the reported existence of multiple conformational isomers of varying folding compactness and varying hydrophobicity (13). Increased hydrophobicity may lead to some increase in ANS binding, which could also explain the mildly increased fluorescence emission observed for BEAT (A) homodimers, which may suffer from suboptimally paired CH3 domains.

In line with the view of structural alteration in the Fc region, thermal stability of the BEAT (B) D84.4Q homodimer Fc was reduced (Fig. 11*A*), whereas the heterodimer Fc was not affected by the D84.4Q substitution. Together with the observed increases in Δ*H*_cal_ (Table 2), this suggested a mechanism of cooperative stabilization where the individual chains are less stable but have proper stability and structure within the heterodimeric assembly, with the latter being confirmed in the crystal structure (Fig. 5). Cooperative stabilization could also be observed for the non–D84.4Q-mutated heterodimer, as well as the KiH heterodimer (Fig. 11, *A* and *C*). The degree of cooperativity, however, was far greater in BEAT compared with KiH, with the D84.4Q-containing heterodimer exhibiting the most cooperativity. DSC analysis of the original BEAT (B) half-antibody Fc revealed a single transition, whereas two transitions were observed for the D84.4Q-mutated half-antibody Fc (Fig. 11*B*). The BEAT (B) D84.4Q homodimer Fc, on the other hand, had a single transition, and the absence of a second transition suggested the existence of interchain interactions in the Fc of the homodimeric complex. Thus, despite disorder and no significant gain in Δ*H*_cal_ in the BEAT (B) D84.4Q homodimeric Fc, some interactions are formed between the halves, keeping the assembly together, and allowing non–disulfide-bonded homodimers to co-migrate by SEC with disulfide-bonded homodimers. For the original BEAT (B) Fc and KiH (H) Fc, a significant cooperative stabilization could be observed upon assembly of half-antibodies into homodimers with increases in *T*_m_ and Δ*H*_cal_ values (Fig. 11, *B* and *D*, and Table 2). Taken together, we propose that assembly of original BEAT (B) and KiH (H) half-antibodies into homodimers comes with a benefit in terms of stability and leads to structurally integer complexes and thus is more likely to occur than formation of homodimers composed of D84.4Q-mutated BEAT (B) half-antibodies, which have little stability to gain. This may be the pivotal factor contributing to the decrease in homodimer formation and the increased HD for D84.4Q-mutated bsAbs. Ultimately, although all interfaces described have stability to gain upon heterodimer formation and promote good HD levels, the BEAT interface has most to gain, which is even greater in the case of the D84.4Q mutation. Such gain in stability may be the key driver that makes BEAT an optimal Hc HD technology. Although the methods described above compellingly support our proposition, they provide an indirect measure of protein structure and assembly, and thus it is worth mentioning that direct methods such as NMR spectroscopy may provide additional insight but were beyond the scope of this work.

The surprising attributes of the BEAT (B) D84.4Q homodimer species gave rise to tremendous advantages for the rapid production of bsAbs at the early discovery stage in which tedious chain ratio optimization for transient transfections is preferably avoided. Not only could we observe a significant increase in the intrinsic HD rate with little contaminating homodimers present, but the unexpected lack of PA-binding site in the BEAT (B) D84.4Q homodimers allows for an optimal purification where the bsAb is the only species with the ability to bind to the affinity matrix (Fig. 6*C*). Ultimately, the D84.4Q BEAT interface ensures high HD levels by counteracting the influence thereon of the binding arms in the Fab–scFv–Fc format.

## Experimental procedures

### Molecular biology

cDNA coding sequences of engineered CH3 domains were designed *in silico* prior to being gene-synthesized at GENEART AG (Regensburg, Germany), as were the WT IgG1 CH1 and hinge, and the IgG1 and IgG3 CH2 and CH3 sequences. cDNA coding sequences for the anti–TAA-1 antibody VH and VL domains (VH2 and VH3 subclasses) were also gene-synthesized, as well as the anti-CD3ε VH/VL domains and the anti–TAA-2 VH/VL domains. Using PCR assembly techniques, Fab-based Hcs were engineered as follows: the VH domain was followed by a human IgG1 CH1 domain, an IgG1 hinge, an IgG1 or IgG3 CH2 domain containing the mutations L234A and L235A (Eu numbering), and an engineered IgG1 or IgG3 CH3 domain containing the BEAT (A) or (B) substitutions, or alternatively the KiH substitutions. Additional substitutions were added or removed by overlapping PCR using appropriate oligonucleotides. In some cases, to abrogate PA binding in the Fab domain, the G65S (Kabat numbering) substitution was added to the anti–TAA-1 VH that was of the VH3-type. For Lcs, VL domains were fused the human CK domain. ScFv-based Hcs were engineered as follows: the VH domain was genetically fused to the VL domain via a (Gly_4_-Ser)_3_ linker and then fused to an IgG1 hinge (starting at Asp^221^, Eu numbering) via a Gly_4_-Thr linker. The hinge was followed by the CH2 and CH3 domains as previously described for the Fab-based Hcs. PCR products were cloned into a modified pcDNA3.1 plasmid (Invitrogen) carrying oriP which is the origin of plasmid replication of Epstein–Barr virus.

### Transient expression and purification

Heterodimeric BEAT antibodies were recombinantly expressed by co-transfecting Hcs based on BEAT (A) and BEAT (B), as well as the cognate Lc into suspension-adapted HEK293-EBNA cells (catalog. no. ATCC-CRL-10852, LGC Standards, Teddington, UK) using polyethyleneimine. For homodimeric antibodies, only one Hc was transfected with the corresponding Lc. The cells were cultured for a period of 4–5 days before harvest. Cell-free culture supernatants were prepared by centrifugation followed by filtration. Homo- and heterodimeric BEAT antibodies were purified by PA, PG, or FcXL chromatography operated under gravity flow (CaptivA^®^ PA resin, Repligen, Waltham, MA, USA; or PG Sepharose 4 Fast Flow, GE Healthcare; or CaptureSelect™ FcXL Affinity Matrix, Thermo Fisher Scientific) before SDS-PAGE analysis (4–12% acrylamide). Where heterodimeric BEAT antibodies free of homodimeric contaminants were required, cell-free supernatants were purified by differential PA chromatography.

### Differential PA chromatography of BEAT antibodies

Cell-free supernatants were loaded onto a 1-ml HiTrap™ MabSelect SuRe™ PA column pre-equilibrated in 0.2 m citrate-phosphate buffer, pH 6, and operated on an ÄKTApurifier™ chromatography system (both from GE Healthcare) at a flow rate of 1 ml/min. Running buffer was 0.2 m citrate-phosphate buffer, pH 6.0. Wash buffer was 0.2 m citrate-phosphate buffer, pH 5.0. Heterodimer elution was performed using 20 mm sodium acetate buffer, pH 4.1, whereas homodimeric species were unbound (BEAT (A) homodimers) or eluted with 0.1 m glycine, pH 3.5 (BEAT (B) homodimers). Elution was followed by OD reading at 280 nm; relevant fractions were pooled and neutralized with 10% volume of 1 m Tris-HCl, pH 8.0. Fractions were analyzed under non-reduced conditions by SDS-PAGE.

### Heterodimerization assay of BEAT antibodies

The proportions of hetero- to homodimers in the PA- and PG-purified preparations were determined by scanning densitometry analysis of the non-reduced SDS–polyacrylamide gel bands (4–12%, Coomassie staining). Relative ratios of the different gel bands were quantified using a FluorChem SP imaging system (Witec AG, Littau, Switzerland) following the manufacturer's protocol.

### BEAT Fc Q3A-D84.4Q protein preparation, crystallization, and structure determination

DNA constructs for crystallization were prepared as follows: cDNAs coding the engineered CH3 domain of BEAT (A) (IgG3 isotype) with the Q3A substitution and the engineered CH3 domain of BEAT (B) (IgG1 isotype) with the D84.4Q substitution were gene-synthesized. Using PCR assembly techniques, each chain had their respective engineered CH3 domain cDNA coding sequence fused downstream of a human IgG1 hinge (DKTHTCPPCP) and IgG3 CH2 constant domain for BEAT (A) and IgG1 CH2 constant domain for BEAT (B). CH2 domains contained the L234A and L235A mutations. A polyhistidine sequence was fused at the C terminus of the BEAT (A) chain, although ultimately it was not required for the purification. Finally, the BEAT (A) and BEAT (B) chain coding DNA sequences were ligated into independent vectors and co-expressed in HEK293-EBNA cells as described above. BEAT Fc Q3A-D84.4Q was purified in a single PA purification step because only the heterodimer bound to the resin: BEAT (A) homodimers were of the IgG3 isotype and thus did not bind PA, BEAT (B) homodimers did not bind PA because of binding abrogation as a result of the D84.4Q substitution. The filtered cell-culture supernatant was applied to CaptivA® PA resin by means of gravity flow. After loading, the column was washed with 0.2 m citrate-phosphate buffer, pH 6.0. Heterodimer elution was performed using 20 mm sodium acetate, pH 3.8. Elution was followed by OD reading at 280 nm; relevant fractions were pooled and neutralized with 10% volume of 1 m Tris-HCl, pH 8.0. The purified BEAT Fc Q3A-D84.4Q was dialyzed against PBS, pH 7.4 (10010023, Gibco, Thermo Fisher Scientific). Crystallization trials and structure determination were performed at Crelux GmbH (Martinsried, Germany). In brief, crystals of BEAT Fc Q3A-D84.4Q were obtained using hanging-drop vapor-diffusion setups after reductive methylation of the protein (Hampton Research reductive alkylation kit, catalog. no. HR2-434, Hampton Research, Aliso Viejo, CA, USA). 0.7 µl of BEAT Fc Q3A-D84.4Q solution (12.0 mg/ml in 10 mm HEPES, 0.1 m NaCl, 1 mm EDTA, pH 8.0) were mixed with 0.7 µl of reservoir solution (33% (w/v) PEG1500) and equilibrated at 20 °C over 0.2 ml of reservoir solution. Well-diffracting crystals appeared within 1 day and grew over 3–4 days to full size. A complete data set of a BEAT Fc Q3A-D84.4Q crystal was collected at the European Synchrotron Radiation Facility radiation source (id30a1, Grenoble, France). Molecular replacement was performed using the previously solved BEAT Fc structure (PDB accession code 5M3V) as a search model (30). Several rounds of alternating manual rebuilding and refinement with REFMAC5 (31) resulted in the final model.

### Antibody modeling

Homology modeling was performed using the YASARA software (32). Interdomain interactions were analyzed using the online PIC server (RRID:SCR_018574) (33). A homology model of the BEAT (B) D84.4Q CH3 homodimer was built based on the original BEAT Fc structure with PDB code 5M3V.

### SPR analyses of BEAT antibodies

The experiments were performed on a Biacore 2000 or Biacore T200 instrument (GE Healthcare) at room temperature. Data fitting was performed using the BIA evaluation software v4.1 or the Biacore T200 evaluation software, version 3.0 (both from GE Healthcare). Human FcR extracellular domains were cloned, expressed, and purified in-house. *K_D_* values for FcRn (UniProt P55899 and P61769) were measured via direct covalent coupling of the antibody onto a CM5 sensor chip. Dissociation was monitored for 2 min. Regeneration was performed with HBS-EP+, pH 7.4 (GE Healthcare). The data were processed using the steady-state fitting model. HBS-EP+ buffer adjusted to pH 6.0 was used as running buffer. Binding of FcɣR1a (UniProt P12314) was tested via PG capture. ∼100 RUs of antibody were immobilized. FcγR1a was injected at 100 nm. Running buffer was HBS-EP+, pH 7.4. Regeneration was with 10 mm glycine buffer, pH 1.5.

### Preparative gel filtration

Experiments were performed on an ÄKTApurifier™ system. The column was Superdex 200 10/300 GL increase (GE Healthcare) calibrated with a high- and low molecular-mass calibration kit (GE Healthcare). Running buffer was PBS, pH 7.4 (Gibco). Flow was at 0.5 ml/min. Injection volume did not exceed 2% of the column volume (0.3–0.5 ml). Fractions of 0.5 ml were collected and analyzed by SDS-PAGE.

### Differential scanning calorimetry

Measurements were carried out on a MicroCal VP-Capillary differential scanning calorimeter (Malvern Instruments). The cell volume was 0.128 ml, the heating rate was 1 °C/min, and the excess pressure was kept at 64 p.s.i. All protein samples were tested at a concentration of 1.0–1.5 mg/ml in PBS, pH 7.4. The partial molar heat capacities and melting curves were analyzed using standard procedures. Thermograms were baseline-corrected and concentration-normalized before being further analyzed using a non–two-state model in the software Origin, version 7.0 (supplied by Malvern Instruments).

### ANS fluorescence

Protein samples (3.5 μm) in PBS, pH 7.4, were mixed with 200 μm ANS (Sigma) prepared in the same buffer and left to equilibrate for 20 min at room temperature. The samples were transferred to a clear 396-well plate with 80 µl/well (12566625; Thermo Fisher Scientific). Fluorescence emission spectra were recorded on a Synergy Neo plate reader (BioTek, Luzern, Switzerland). Excitation was at 370 nm, and emission was recorded between 400 and 700 nm in steps of 1 nm. The measurements were performed at 20 °C. The fluorescence signal from the buffer alone with 20 μm ANS was subtracted.

## Data availability

The atomic coordinates and structure factors (PDB code 6G1E) have been deposited in the Protein Data Bank. All other data are contained within the article.

## Supplementary Material

Supporting Information
