# Supplementary material for: A single mutation increases heavy-chain heterodimer assembly of bispecific antibodies by inducing structural disorder in one homodimer species
Source: J Biol Chem. 2020 May 13;295(28):9392–408. doi: 10.1074/jbc.RA119.012335 (PMC7363136; doi:10.1074/jbc.RA119.012335)
Supplement: Supporting Information [file supp_295_28_9392__index.html]

A single mutation increases heavy chain heterodimer assembly of bispecific antibodies by inducing structural disorder in one homodimer species — Induced disorder in homodimer increases bispecific assembly — A single mutation increases heavy-chain heterodimer assembly of bispecific antibodies by inducing structural disorder in one homodimer species — Induced disorder in homodimer increases bispecific assembly — Supporting Information 

# A single mutation increases heavy-chain heterodimer assembly of bispecific antibodies by inducing structural disorder in one homodimer species

## Supporting Information

- Supporting Information (to be published online) - Source File for Supplemental Data - to be published
