# Supplementary material for: A single mutation increases heavy-chain heterodimer assembly of bispecific antibodies by inducing structural disorder in one homodimer species
Source: J Biol Chem. 2020 May 13;295(28):9392–408. doi: 10.1074/jbc.RA119.012335 (PMC7363136; doi:10.1074/jbc.RA119.012335)
Supplement: Supporting Information [file supp_RA119.012335_157747_2_supp_527301_qw0wwv.docx]

**Supplemental Data for**

**A single mutation increases heavy chain heterodimer assembly of bispecific antibodies by inducing structural disorder in one homodimer species**

Cian Stutz and Stanislas Blein^*^

From the Department of Antibody Engineering, Ichnos Sciences S.A., Biopôle Lausanne – Epalinges, Bâtiment SE-B, Route de la Corniche 5, 1066 Epalinges, Switzerland

^*^To whom correspondence should be addressed: Email: [stanislas.blein@ichnossciences.com](mailto:stanislas.blein@ichnossciences.com), Tel: +41 21 546 06 00.

**TABLE S1**

Human FcRn binding affinities of the anti-TAA-1 based BEAT and KiH homo- and heterodimers. Average KD values ± standard error (SEM) are shown. Measurements were performed in triplicate.

| **Construct** | **Isotype*** | **Average KD ± SEM** |
| --- | --- | --- |
| Anti-TAA-1 BEAT (A) homodimer | 1133 | 460 ± 11 nM |
| Anti-TAA-1 BEAT (B) homodimer | 1111 | 440 ± 20 nM |
| Anti-TAA-1 BEAT (B) D84.4Q homodimer | 1111 | No binding |
| Anti-TAA-1 BEAT heterodimer | 1133-1111 | 398 ± 15 nM |
| Anti-TAA-1 BEAT D84.4Q heterodimer | 1133-1111 | 350 ± 7 nM |
| Anti-TAA-1 KiH (K) homodimer | 1133 | 506 ± 14 nM |
| Anti-TAA-1 KiH (H) homodimer | 1111 | 1121 ± 17 nM |
| Anti-TAA-1 KiH heterodimer | 1133-1111 | 486 ± 8 nM |
| *Numerals correspond to the IgG isotype subclass of each domain in the order of: CH1/hinge/CH2/CH3 | | |
